# The *Candida albicans* Histone Acetyltransferase Hat1 Regulates Stress Resistance and Virulence via Distinct Chromatin Assembly Pathways

**DOI:** 10.1371/journal.ppat.1005218

**Published:** 2015-10-16

**Authors:** Michael Tscherner, Florian Zwolanek, Sabrina Jenull, Fritz J. Sedlazeck, Andriy Petryshyn, Ingrid E. Frohner, John Mavrianos, Neeraj Chauhan, Arndt von Haeseler, Karl Kuchler

**Affiliations:** 1 Department for Medical Biochemistry, Medical University of Vienna, Max F. Perutz Laboratories, Campus Vienna Biocenter, Vienna, Austria; 2 Center for Integrative Bioinformatics Vienna, Max F. Perutz Laboratories, University of Vienna, Medical University of Vienna, Campus Vienna Biocenter, Vienna, Austria; 3 Public Health Research Institute, New Jersey Medical School - Rutgers, The State University of New Jersey, Newark, New Jersey, United States of America; University of Rochester, UNITED STATES

## Abstract

Human fungal pathogens like *Candida albicans* respond to host immune surveillance by rapidly adapting their transcriptional programs. Chromatin assembly factors are involved in the regulation of stress genes by modulating the histone density at these loci. Here, we report a novel role for the chromatin assembly-associated histone acetyltransferase complex NuB4 in regulating oxidative stress resistance, antifungal drug tolerance and virulence in *C*. *albicans*. Strikingly, depletion of the NuB4 catalytic subunit, the histone acetyltransferase Hat1, markedly increases resistance to oxidative stress and tolerance to azole antifungals. Hydrogen peroxide resistance in cells lacking Hat1 results from higher induction rates of oxidative stress gene expression, accompanied by reduced histone density as well as subsequent increased RNA polymerase recruitment. Furthermore, *hat1*Δ/Δ cells, despite showing growth defects *in vitro*, display reduced susceptibility to reactive oxygen-mediated killing by innate immune cells. Thus, clearance from infected mice is delayed although cells lacking Hat1 are severely compromised in killing the host. Interestingly, increased oxidative stress resistance and azole tolerance are phenocopied by the loss of histone chaperone complexes CAF-1 and HIR, respectively, suggesting a central role for NuB4 in the delivery of histones destined for chromatin assembly via distinct pathways. Remarkably, the oxidative stress phenotype of *hat1*Δ/Δ cells is a species-specific trait only found in *C*. *albicans* and members of the CTG clade. The reduced azole susceptibility appears to be conserved in a wider range of fungi. Thus, our work demonstrates how highly conserved chromatin assembly pathways can acquire new functions in pathogenic fungi during coevolution with the host.

## Author Summary

*Candida albicans* is the most prevalent fungal pathogen infecting humans, causing life-threatening infections in immunocompromised individuals. Host immune surveillance imposes stress conditions upon *C*. *albicans*, to which it has to adapt quickly to escape host killing. This can involve regulation of specific genes requiring disassembly and reassembly of histone proteins, around which DNA is wrapped to form the basic repeat unit of eukaryotic chromatin—the nucleosome. Here, we discover a novel function for the chromatin assembly-associated histone acetyltransferase complex NuB4 in oxidative stress response, antifungal drug tolerance as well as in fungal virulence. The NuB4 complex modulates the induction kinetics of hydrogen peroxide-induced genes. Furthermore, NuB4 negatively regulates susceptibility to killing by immune cells and thereby slowing the clearing from infected mice *in vivo*. Remarkably, the oxidative stress resistance seems restricted to *C*. *albicans* and closely related species, which might have acquired this function during coevolution with the host.

## Introduction

Eukaryotic chromatin is densely packed with the nucleosome being its basic repeating unit [1]. This structure represents a barrier for enzymes reading or modifying genomic DNA. Thus, disassembly and reassembly of histones, the core components of nucleosomes, is essential for various biological processes, including transcription, replication and DNA repair [2–7]. Two key players in the deposition of newly synthesized histones into chromatin are type B histone acetyltransferases (HATs) and histone chaperones. Type B HATs specifically acetylate free histones immediately after synthesis and show at least partial cytoplasmic localization. Hat1 was the first type B HAT identified and was found conserved throughout the eukaryotic kingdom [8,9]. Together with the Hat2 subunit (RbAp46/48 in higher eukaryotes), Hat1 acetylates histone H4 at lysine 5 and 12 in *Saccharomyces cerevisiae* and *Candida albicans* [9,10]. After binding of an additional subunit in the nucleus, the so-called NuB4 complex is formed, which is responsible for histone deposition at sites of DNA damage [11,12]. Rtt109 is another fungal-specific Type B HAT involved in DNA damage repair and associated histone deposition by acetylating free histone H3 at lysine 56 [2,13]. Interestingly, telomeric silencing is also defective in *S*. *cerevisiae* and *Schizosaccharomyces pombe hat1*Δ mutants [14,15]. Thus, Hat1 appears to be also involved in the generation or maintenance of repressive chromatin structures.

Hat1 operates with various histone chaperones [16–18]. This class of proteins is able to bind histones thereby avoiding unspecific interactions with DNA and facilitating correct incorporation into nucleosomes [19]. Two main chromatin assembly pathways include the hallmark histone chaperone complexes CAF-1 and HIR. While CAF-1 is well known to function in replication-coupled chromatin assembly, recent reports indicate also a role in transcription regulation [20–22]. In contrast, HIR is involved in replication-independent chromatin assembly and acts as a repressor of histone genes outside of S-phase [23]. Of note, Hat1 was found in complexes together with CAF-1 or HIR, thus suggesting a central role in the delivery of newly synthesized histones for incorporation via distinct pathways [16,18].

The opportunistic human fungal pathogen *Candida albicans* is the most frequent cause of *Candida*-derived invasive infections [24]. *Candida* spp. can cause diseases ranging from chronic mucocutaneous to life-threatening systemic infections in immunocompromised patients. *C*. *albicans* belongs to the CTG clade, a group of closely related species which translate the CUG codon as serine instead of leucine [25]. During the infection process, *C*. *albicans* encounters various environmental stress conditions, including reactive oxygen species (ROS) produced by innate immune cells such as macrophages, dendritic cells or neutrophils dedicated to kill invading pathogens [26]. Furthermore, treatment with antifungal agents such as azoles, which inhibit fungal ergosterol biosynthesis and are commonly used to treat *Candida* infections, also represents severe stress to the pathogen [27]. At least two major mechanisms are therefore essential for *C*. *albicans* to be able to survive under these conditions: the fungus has to be able to repair damaged cellular components efficiently, and it has to respond rapidly by adapting transcriptional programs to counteract immune defense [28–32]. Changes in chromatin structure are involved in both mechanisms. Disassembly and reassembly of histones is required for efficient repair of DNA damage [2,7]. Furthermore, transcriptional modulation is intimately linked to the histone density at the corresponding loci [6,33–35]. Interestingly, several studies reported functions of chromatin-remodeling factors in the regulation of stress-responsive genes [33,36–38]. For instance, incorporation of histones into chromatin is particularly important for efficient gene repression, as the increase of histone density impairs binding of activators and inhibits RNA polymerase progression [6,39,40].

We have recently shown that *C*. *albicans* NuB4 complex is required for efficient repair of DNA damage resulting from endogenous or exogenous impact [10]. Here, we report a novel function for NuB4 in the negative regulation of oxidative stress resistance and azole tolerance. The Hat1 component of NuB4 acts in concert with distinct chromatin assembly pathways, which is independent of its conserved role in DNA damage repair. We show that the loss of the NuB4 complex markedly increases oxidative stress resistance through an accelerated induction of oxidative stress genes. Furthermore, this renders the pathogen resistant to killing by innate immune cells and promotes persistence of a *hat1*Δ/Δ mutant in a murine infection model. Interestingly, loss of Cac2, a subunit of the CAF-1 histone chaperone complex, mimics the oxidative stress resistance phenotype of *hat1*Δ/Δ cells. Furthermore, transcriptional profiling by RNA-Seq confirms overlapping functions of NuB4 and CAF-1 in *C*. *albicans*. Moreover, we also discover a novel role for NuB4 requiring the HIR complex for the negative regulation of azole tolerance in *C*. *albicans*. Interestingly, whereas the oxidative stress phenotype of *hat1*Δ/Δ cells has exclusively evolved in the *Candida* CTG clade, the reduced azole susceptibility seems to be conserved in a wider range of fungal species. Thus, our work demonstrates how highly conserved chromatin assembly pathways can acquire new functions, as for example in pathogenic fungi during coevolution with the host. Thus, host-specific immune defense mechanisms can act as drivers of evolutionary adaptation, enabling pathogens to cope with specific stress conditions.

## Results

### Deletion of NuB4 components increases H_2_O_2_ resistance and azole tolerance

**Fig 1:**
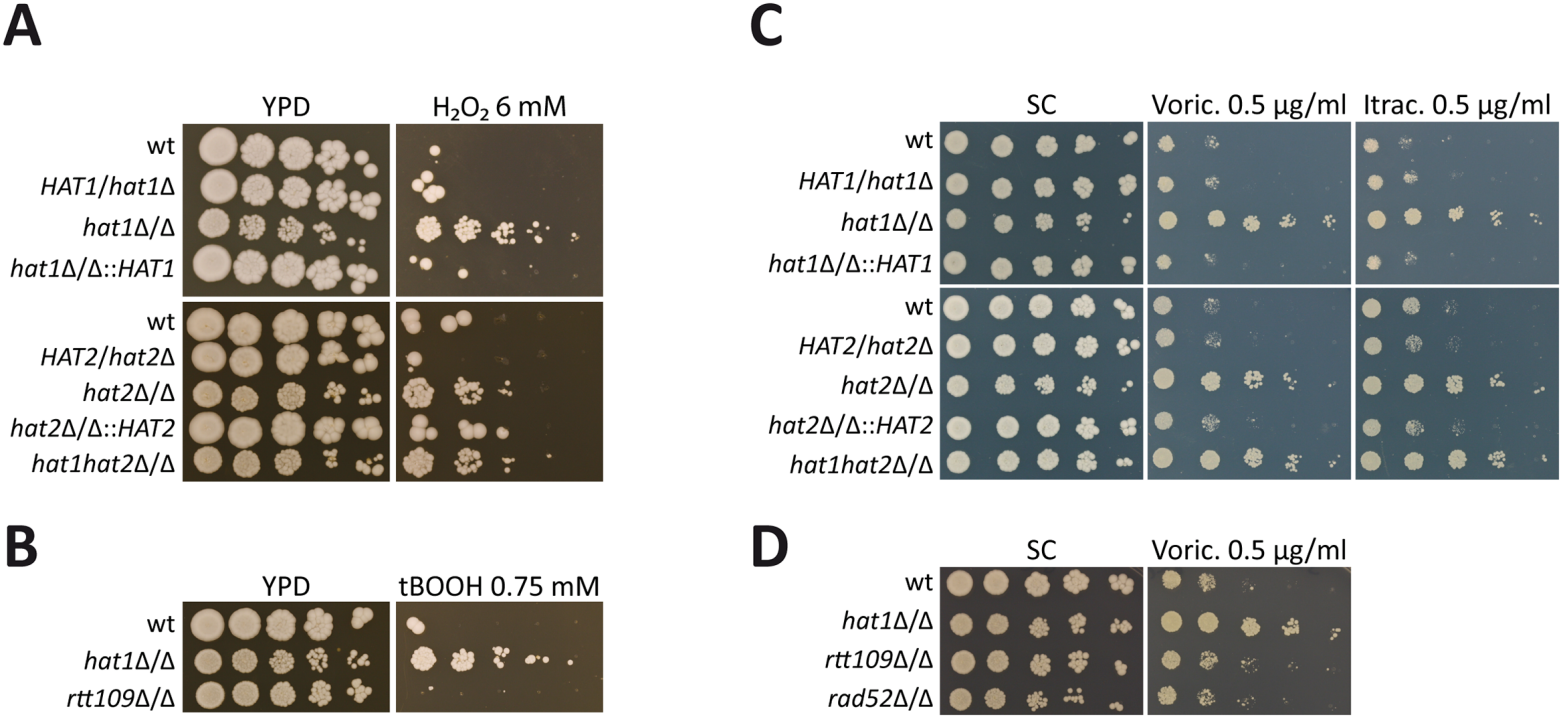
Deletion of *HAT1* and *HAT2* increases oxidative stress resistance and azole tolerance. (A) Cells lacking Hat1 or Hat2 show increased resistance to H_2_O_2_. Lack of both genes mimics the corresponding single deletion strains. (B) Deletion of *HAT1* increases resistance to *tert*-butyl hydroperoxide (tBOOH). Lack of Rtt109 does not affect tBOOH sensitivity. (C) Loss of Hat1 causes reduced susceptibility to voriconazole (Voric.) and itraconazole (Itrac.). Deletion of *HAT2* or *HAT1* and *HAT2* mimics loss of Hat1. (D) Deletion of *RTT109* or *RAD52* does not increase voriconazole tolerance. (A-D) Fivefold serial dilutions of the indicated strains were spotted on agar plates containing the indicated substances and pictures were taken after incubation at 30°C for 3 days.

In a previous study, we identified the *C*. *albicans* NuB4 complex being essential for efficient repair of both exogenous and endogenous DNA damage [10]. *C*. *albicans* DNA damage repair mutants show increased susceptibility to ROS produced by immune cells [30]. Therefore, we asked if inactivation of the NuB4 complex would render this pathogen hypersusceptible to H_2_O_2_. Surprisingly, however, deletion of *HAT1*, *HAT2* or both genes increased the resistance to H_2_O_2_ as determined by spot dilution assays (Fig 1A). Importantly, reintegration of *HAT1* or *HAT2* at its endogenous locus fully restored the wild-type phenotype (Fig 1A).

Due to this unexpected resistance phenotype, we subjected the *hat1*Δ/Δ mutant to phenotypic analysis by applying a set of different stress conditions including cell wall stress (Calcofluor White, Congo Red), osmotic stress (NaCl), oxidative stress (*tert*-Butyl hydroperoxide (tBOOH), diamide), heavy metal stress (CdCl_2_), as well as antifungal drugs (Voriconazole, Itraconazole, Amphotericin B). For most conditions, we did not observe any difference between the wild-type and the *hat1*Δ/Δ strain (S1A Fig). However, lack of Hat1 markedly increased resistance to the oxidizing agents tBOOH and diamide, indicating a specific role for Hat1 in the regulation of oxidative stress resistance (Fig 1B and S1B Fig).

Interestingly, we also observed that deletion of *HAT1* increased tolerance to different azole drugs (Fig 1C). Azoles represent a widely used class of antifungals targeting the lanosterol 14-α-demethylase, thereby blocking fungal ergosterol biosynthesis. Furthermore, deletion of *HAT2* or *HAT1* and *HAT2* mimicked deletion of *HAT1* and reintegration of both genes at their endogenous loci fully restored the wild-type phenotype (Fig 1C). To confirm that the observed resistance phenotypes are independent of the NuB4 function in DNA damage repair, we determined sensitivities of different DNA damage repair mutants to H_2_O_2_ and voriconazole. Importantly, neither deletion of the gene encoding the histone H3 specific acetyltransferase Rtt109 nor the absence of the repair protein Rad52 yielded in comparable oxidative stress or voriconazole phenotypes (Fig 1B, 1D and S1C Fig). Hence, not all defects in DNA repair lead to oxidative stress resistance. These data suggest that Hat1 has an additional and novel function in *C*. *albicans* in the regulation of oxidative stress resistance and antifungal drug tolerance.

### Inactivation of chromatin assembly pathways mimics lack of Hat1

Hat1 is involved in the deposition of histone H3–H4 dimers at sites of DNA damage and in heterochromatic regions in different species [14,41–43]. Different histone chaperones are responsible for the incorporation of histones into nucleosomes via distinct chromatin assembly pathways [20,44–47]. We hypothesized that a defect in chromatin assembly is causing the observed resistance phenotypes. Therefore, we created deletion mutants of a set of genes encoding histone chaperones or histone chaperone complex components known to interact or copurify with Hat1 or to regulate gene expression in other species (Table 1). Genes were deleted in wild-type cells as well as in a *hat1*Δ/Δ background and sensitivities to H_2_O_2_ and voriconazole were tested.

**Table 1:** Selected histone chaperone genes for deletion in *C*. *albicans*.

Gene Name	ORF number	Complex	Deleted	Reference
*CAC2*	orf19.6670	CAF-1	+	[16,48]
*HIR1*	orf19.2099	HIR/HIRA	+	[18]
*RTT106*	orf19.1177		+	[38,49]
*ASF1*	orf19.3715		-	[16,17]
*SPT16*	orf19.2884	FACT	-	[50]
*SPT6*	orf19.7136		+	[33,35]

Histone chaperone genes chosen for deletion are listed. + indicates successful deletion;—indicates that no deletion mutant was obtained.

**Fig 2:**
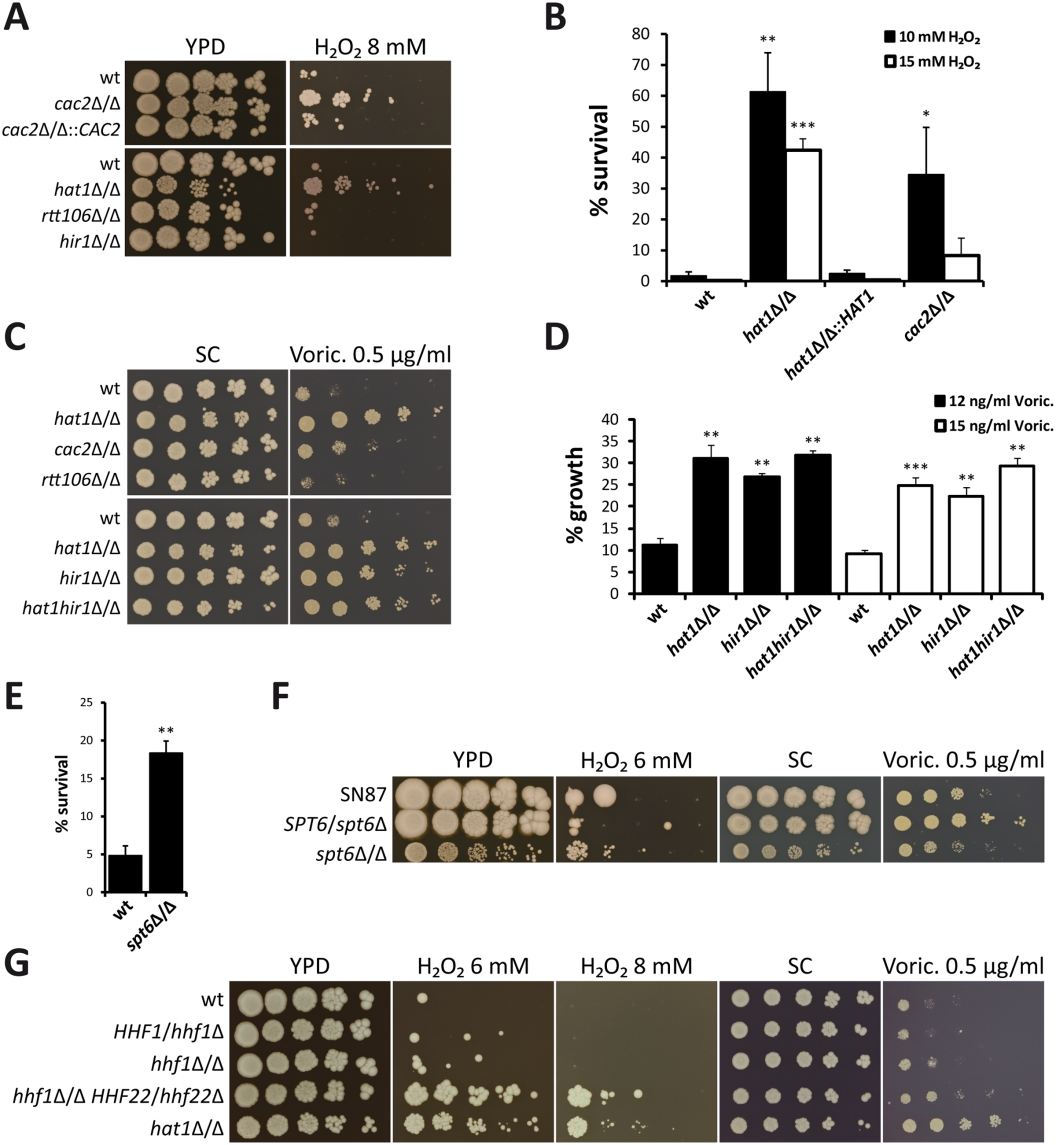
Lack of histone chaperones mimics deletion of *HAT1*. (A) Loss of Cac2 increases H_2_O_2_ resistance. Deletion of *RTT106* or *HIR1* does not affect susceptibility to hydrogen peroxide. Fivefold serial dilutions of the indicated strains were spotted on agar plates containing the indicated substances and pictures were taken after incubation at 30°C for 3 days. (B) Deletion of *HAT1* or *CAC2* increases survival to transient hydrogen peroxide treatment. Exponentially growing cells were treated with the indicated concentrations of H_2_O_2_ for 2 hours. Cells were plated and colonies counted after 3 days of incubation on YPD plates at 30°C to determine viability. Data are shown as mean + SD from three independent experiments. (C) Deletion of *HIR1* reduces voriconazole (Voric.) susceptibility. The *hat1hir1*Δ/Δ double deletion strain mimics lack of Hat1. Loss of Cac2 has only a minor effect and deletion of *RTT106* does not alter azole susceptibility. Experiment was performed as described in (A). (D) Increased azole tolerance of *hat1*Δ/Δ, *hir1*Δ/Δ and *hat1hir1*Δ/Δ was confirmed using a liquid growth inhibition assay. Logarithmically growing cells were diluted into medium containing the indicated concentrations of voriconazole (Voric.) and incubated at 30°C for 18 hours. OD_600_ was determined and growth inhibition relative to untreated samples was calculated. Data are shown as mean + SD from three independent experiments. (E) Lack of Spt6 reduces H_2_O_2_ susceptibility. Experiment was performed as described in (B). Cells were treated with 10 mM H_2_O_2_. Data are shown as mean + SD from two independent experiments. (F) Deletion of *SPT6* increases H_2_O_2_ resistance and azole tolerance. Fivefold serial dilutions of the indicated strains were spotted on agar plates containing the indicated substances and pictures were taken after incubation at 30°C for 5 days. (G) Reduction of histone gene dosage decreases H_2_O_2_ and azole susceptibility. Experiment was performed as described in (A). (B, D, E) *P<0.05, **P<0.01 and ***P<0.001 relative to the corresponding wild-type (Student's t-test).

Strikingly, lack of *CAC2*, a subunit of the CAF-1 histone chaperone complex, also strongly increased resistance to H_2_O_2_ and tBOOH (Fig 2A and S1D Fig). Furthermore, a quantification of H_2_O_2_ resistance by determination of the survival rate in liquid culture confirmed this result (Fig 2B). Interestingly however, spot dilution assays suggested a minor effect on azole susceptibility of *cac2*Δ/Δ cells (Fig 2C). By contrast, deletion of *HIR1*, a component of the HIR histone chaperone complex, dramatically increased tolerance to voriconazole, but did not alter H_2_O_2_ susceptibility (Fig 2A and 2C). Importantly, *HAT1* and *HIR1* are epistatic, since a double deletion strain failed to show increased azole resistance when compared to the corresponding single deletions based on spot dilution assays and growth inhibition in liquid culture (Fig 2C and 2D).

Deletion of *RTT106* did not alter susceptibility to H_2_O_2_ and voriconazole (Fig 2A and 2C). Unfortunately, we and others were unable to construct homozygous deletion mutants for *ASF1* and *SPT16*, indicating that they might be essential [51,52]. We also included the *C*. *albicans* mutant lacking the histone chaperone Spt6 [53]. Similar to *HAT1*, deletion of *SPT6* increased H_2_O_2_ resistance (Fig 2E and 2F). However, a reliable and accurate determination of azole sensitivities of the homozygous *spt6*Δ/Δ mutant was not possible due to its pronounced slow-growth phenotype. Of note, deletion of one *SPT6* allele also decreased susceptibility to voriconazole (Fig 2F).

In a previous study, we showed that reducing histone H4 gene dosage mimics the genetic deletion of *HAT1* with respect to sensitivity to genotoxic stress [10]. Furthermore, a common set of genes is differentially regulated upon depletion of histone H4 and deletion of *CAC2* in *S*. *cerevisiae* [37]. Thus, we determined the effect of histone H4 reduction on oxidative stress and azole susceptibility. Interestingly, strains harboring a single copy of the histone H4 gene remaining also displayed increased H_2_O_2_ resistance and slightly elevated tolerance to voriconazole (Fig 2G). In summary, these data indicate that defects in distinct *C*. *albicans* chromatin assembly pathways can alter susceptibilities to H_2_O_2_ and voriconazole, thus regulating oxidative stress response and tolerance to azole antifungals, respectively.

### Oxidative stress resistance upon loss of Hat1 is restricted to CTG clade species

**Fig 3:**
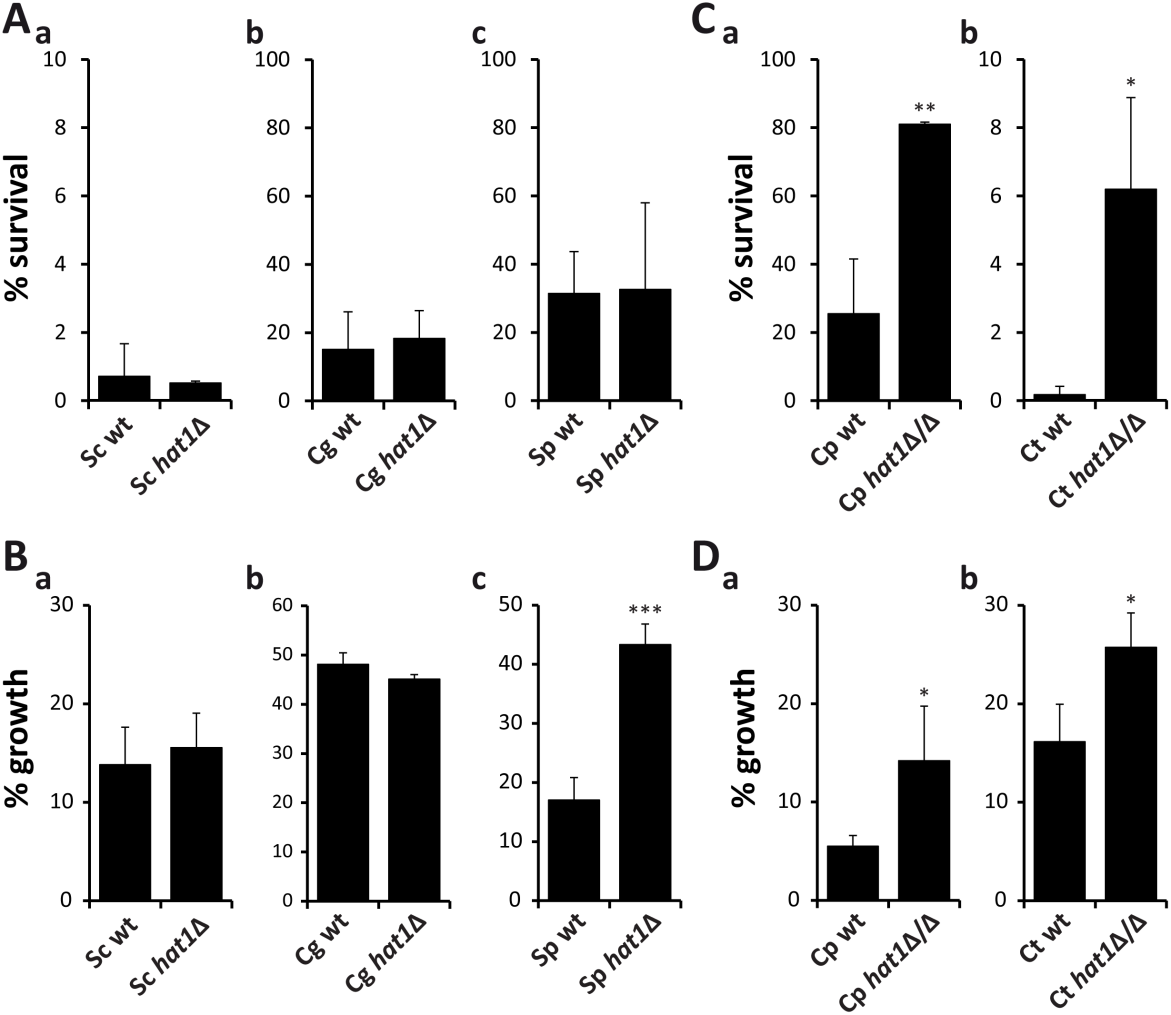
Resistance phenotypes caused by loss of Hat1 are specific for *C*. *albicans*. (A) Deletion of *HAT1* in *S*. *cerevisiae* (a), *C*. *glabrata* (b) and *S*. *pombe* (c) has no effect on H_2_O_2_ resistance. Exponentially growing cells were treated with 5 mM (a), 50 mM (b) or 20 mM (c) H_2_O_2_ for 2 hours. Cells were plated and colonies counted after 3 days of incubation on YPD plates at 30°C to determine viability. Data are shown as mean + SD from three independent experiments. (B) Lack of Hat1 in *S*. *cerevisiae* (a) and *C*. *glabrata* (b) does not increase azole tolerance. Deletion of Hat1 in *S*. *pombe* reduces susceptibility to voriconazole (c). Logarithmically growing cells were diluted into medium containing 150 ng/ml (a), 1000 ng/ml (b) or 800 ng/ml (c) voriconazole and incubated at 30°C for 24 hours. OD_600_ was determined and growth inhibition relative to untreated samples was calculated. Data are shown as mean + SD from three independent experiments. (C) *C*. *parapsilosis* (a) and *C*. *tropicalis* (b) *hat1*Δ/Δ cells show increased resistance to H_2_O_2_. Experiment was performed as described in (A). H_2_O_2_ concentrations were 50 mM (a) and 20 mM (b). (D) Loss of Hat1 in *C*. *parapsilosis* (a) and *C*. *tropicalis* (b) reduces susceptibility to voriconazole. Experiment was performed as described in (B). For *C*. *parapsilosis* cells were incubated for 41 hours prior to OD_600_ measurement. Voriconazole concentrations were 50 ng/ml (a) and 200 ng/ml (b). (A-D) *P<0.05, **P<0.01 and ***P<0.001 relative to the corresponding wild-type (Student's t-test).

Next, we wanted to determine if the role of Hat1 in the regulation of oxidative stress resistance and azole tolerance is conserved in other fungal species. Therefore, we analyzed the effect of *HAT1* deletion on H_2_O_2_ and voriconazole susceptibility in the distantly related fungi *Saccharomyces cerevisiae*, *Candida glabrata* and *Schizosaccharomyces pombe*. However, loss of Hat1 did not lead to increased resistance to H_2_O_2_ in any of these species (Fig 3A). Furthermore, lack of Hat1 failed to lower voriconazole sensitivity in *S*. *cerevisiae* and *C*. *glabrata* (Fig 3B), but increased azole tolerance in *S*. *pombe* (Fig 3Bc).

Based on these results, we speculated that Hat1 might have acquired a role in the regulation of oxidative stress resistance only later in evolution. To prove this, we constructed homozygous *HAT1* deletion mutants in the more closely related CTG clade members *Candida parapsilosis* and *Candida tropicalis* and determined their H_2_O_2_ sensitivity. Strikingly, lack of Hat1 decreased the susceptibility to H_2_O_2_ (Fig 3C) and increased tolerance to voriconazole in both species (Fig 3D). Thus, the role of Hat1 in regulating oxidative stress resistance appears specific for *C*. *albicans* and related species within the CTG clade, while the function in azole tolerance is present in a wider range of fungal species.

### Loss of Hat1 primarily affects transcriptional repression

To investigate the effect of *HAT1* deletion during normal growth, and to elucidate the mechanism of oxidative stress resistance in the *hat1*Δ/Δ mutant, we determined transcriptional profiles of cells during the logarithmic growth phase and upon H_2_O_2_ treatment. Therefore, we performed RNA sequencing (RNA-Seq) analysis on cells before and after exposure to 1.6 mM H_2_O_2_ for 30 minutes. Importantly, no loss of viability was observed even after 1 hour treatment at this peroxide concentration (S2A Fig). Furthermore, transcriptional profiles were also determined for the *cac2*Δ/Δ strain, since this mutant also showed an oxidative stress resistance phenotype. In addition, we included *rtt109*Δ/Δ cells as a control, as it mimics lack of Hat1 concerning morphology and accumulation of DNA damages [10,30], yet shows wild-type susceptibility to H_2_O_2_ (S1C Fig).

**Fig 4:**
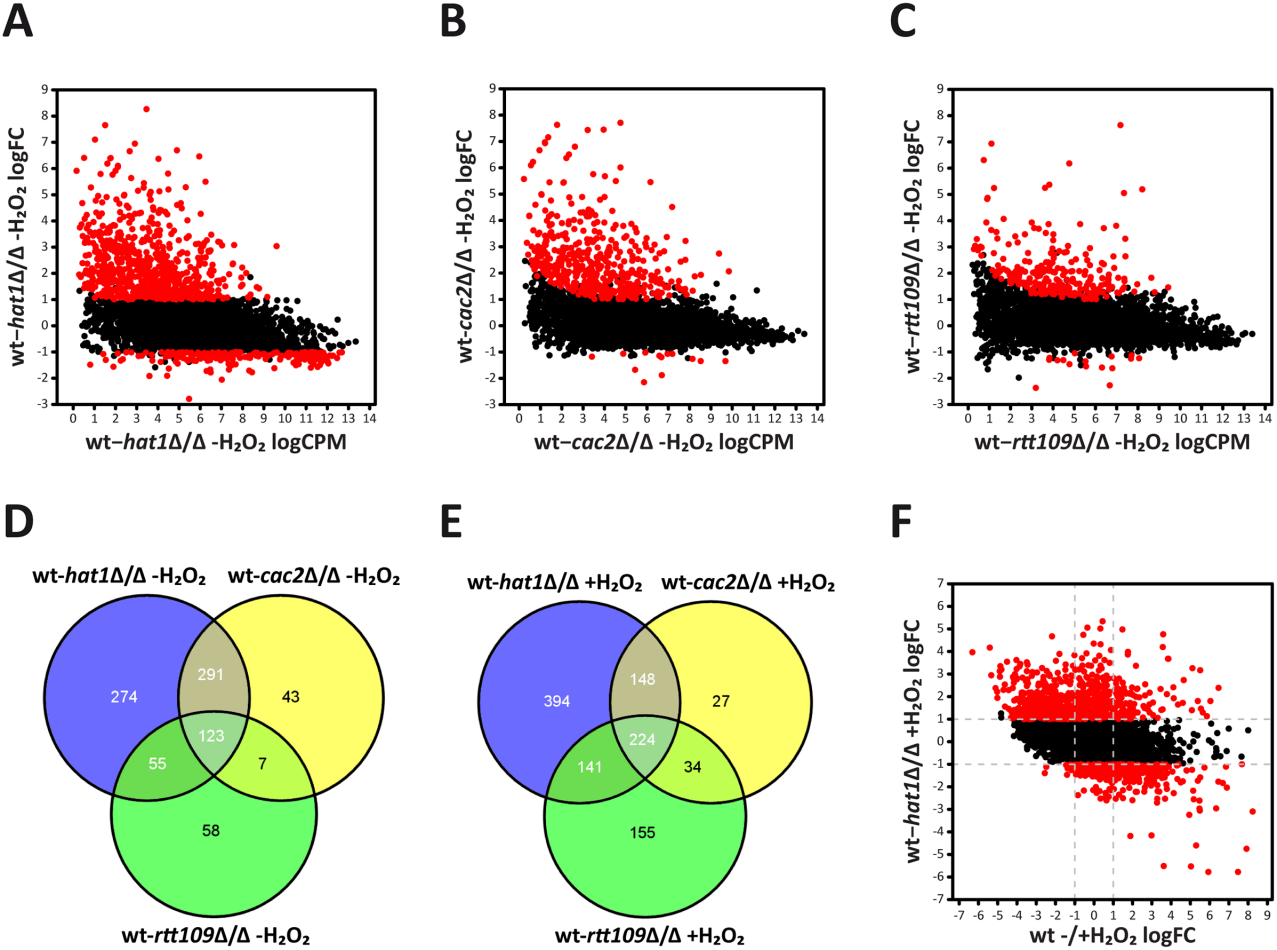
Deletion of *HAT1* primarily leads to upregulation of genes. (A) Lack of Hat1 causes mainly induction of genes in logarithmically growing cells. Each dot corresponds to one protein-coding gene. The fold change in RNA expression between untreated wild-type and *hat1*Δ/Δ cells (y-axis) is plotted against the expression level of each gene in this dataset (x-axis). Differentially expressed genes in the *hat1*Δ/Δ mutant are depicted in red. logCPM: log2 counts per million reads; logFC: log2 fold change; (B+C) Loss of Cac2 or Rtt109 causes almost exclusively upregulation of genes in logarithmically growing cells. Plots were created as described in (A). (D) Venn diagram showing the overlaps of upregulated genes in the *hat1*Δ/Δ, *cac2*Δ/Δ and *rtt109*Δ/Δ mutants in the absence of H_2_O_2_. (E) Venn diagram showing the overlaps of upregulated genes in the *hat1*Δ/Δ, *cac2*Δ/Δ and *rtt109*Δ/Δ mutants upon treatment with H_2_O_2_. (F) H_2_O_2_ repressed genes are upregulated in the *hat1*Δ/Δ mutant upon peroxide treatment. Each dot corresponds to one protein-coding gene. The -fold change in RNA expression between H_2_O_2_ treated wild-type and *hat1*Δ/Δ strains (y-axis) is plotted against the fold change between the wild-type without and with treatment (x-axis). Differentially expressed genes in the *hat1*Δ/Δ mutant are depicted in red. logFC: log2 fold change; (A-F) Differentially regulated genes were defined by a fold change > = 2 and p-value <0.05.

RNA-Seq analysis of logarithmically growing cells showed that genetic removal of *HAT1* primarily upregulates a large number of genes. We found 743 genes at least 2-fold induced and 209 genes 2-fold repressed in the *hat1*Δ/Δ mutant when compared to the wild-type. Furthermore, the amplitude of gene repression was clearly lower when compared to the upregulation of genes, implying that Hat1 exerts primarily repressing rather than activating functions (Fig 4A). Also for the *cac2*Δ/Δ and *rtt109*Δ/Δ mutants, we observed an almost exclusive upregulation of gene expression when compared to the wild-type (Fig 4B and 4C). However, lack of Cac2 or Rtt109 had a less pronounced effect concerning the number of upregulated genes when compared to the loss of Hat1. In the *cac2*Δ/Δ and the *rtt109*Δ/Δ strains, 464 and 243 genes were transcriptionally upregulated, respectively. Comparison of regulated genes in the *hat1*Δ/Δ, the *cac2*Δ/Δ and the *rtt109*Δ/Δ mutants revealed large overlaps between all datasets (Fig 4D) with 123 genes upregulated in all three mutants. Interestingly, the majority of upregulated genes in the *cac2*Δ/Δ mutant were also induced in the *hat1*Δ/Δ strain. As expected, we also detected a large overlap between the *hat1*Δ/Δ and the *rtt109*Δ/Δ strain, since both mutants share functions in DNA damage repair and cell morphology [10,30].

Following H_2_O_2_ treatment, downregulated genes in the *hat1*Δ/Δ mutant increased to 459 when compared to the wild-type, although under these conditions the majority of differentially regulated genes remain up (907). The comparison of upregulated genes in the *hat1*Δ/Δ, the *cac2*Δ/Δ and the *rtt109*Δ/Δ mutants again revealed large overlaps between the three datasets (Fig 4E). Similar to the untreated condition, most genes with higher expression levels in the *cac2*Δ/Δ strain were also upregulated in the *hat1*Δ/Δ mutant, suggesting redundant functions of Hat1 and Cac2 in the regulation of gene expression. Furthermore, we again detected a large overlap between the *hat1*Δ/Δ and the *rtt109*Δ/Δ strains, and a common set of genes upregulated in all three mutants (Fig 4E). In addition, a large fraction of upregulated genes (449) in the *hat1*Δ/Δ mutant were repressed in the wild-type upon treatment with H_2_O_2_, indicating a defect in repression due to the lack of Hat1 (Fig 4F). However, we also detected a smaller set of upregulated genes (104) in the *hat1*Δ/Δ strain that were induced in the wild-type. Thus, lack of Hat1 primarily leads to upregulation of genes in logarithmically growing cells, as well as upon H_2_O_2_ treatment indicating a repressive function of Hat1 in *C*. *albicans*.

In addition to protein-coding genes, we also analyzed the expression of non-coding elements, including tRNAs and small RNAs. Without treatment, most non-coding RNAs were not differentially expressed in the *hat1*Δ/Δ mutant (S1 Table). Out of 193 non-coding RNAs, 64 were repressed more than two-fold in wild-type cells upon H_2_O_2_ treatment (S2B Fig). Interestingly, in the *hat1*Δ/Δ mutant, 20 of these non-coding RNAs were upregulated at least 2-fold when compared to the wild-type, again indicating impaired repression upon loss of Hat1.

### Lack of Hat1 affects specific functional gene groups

**Fig 5:**
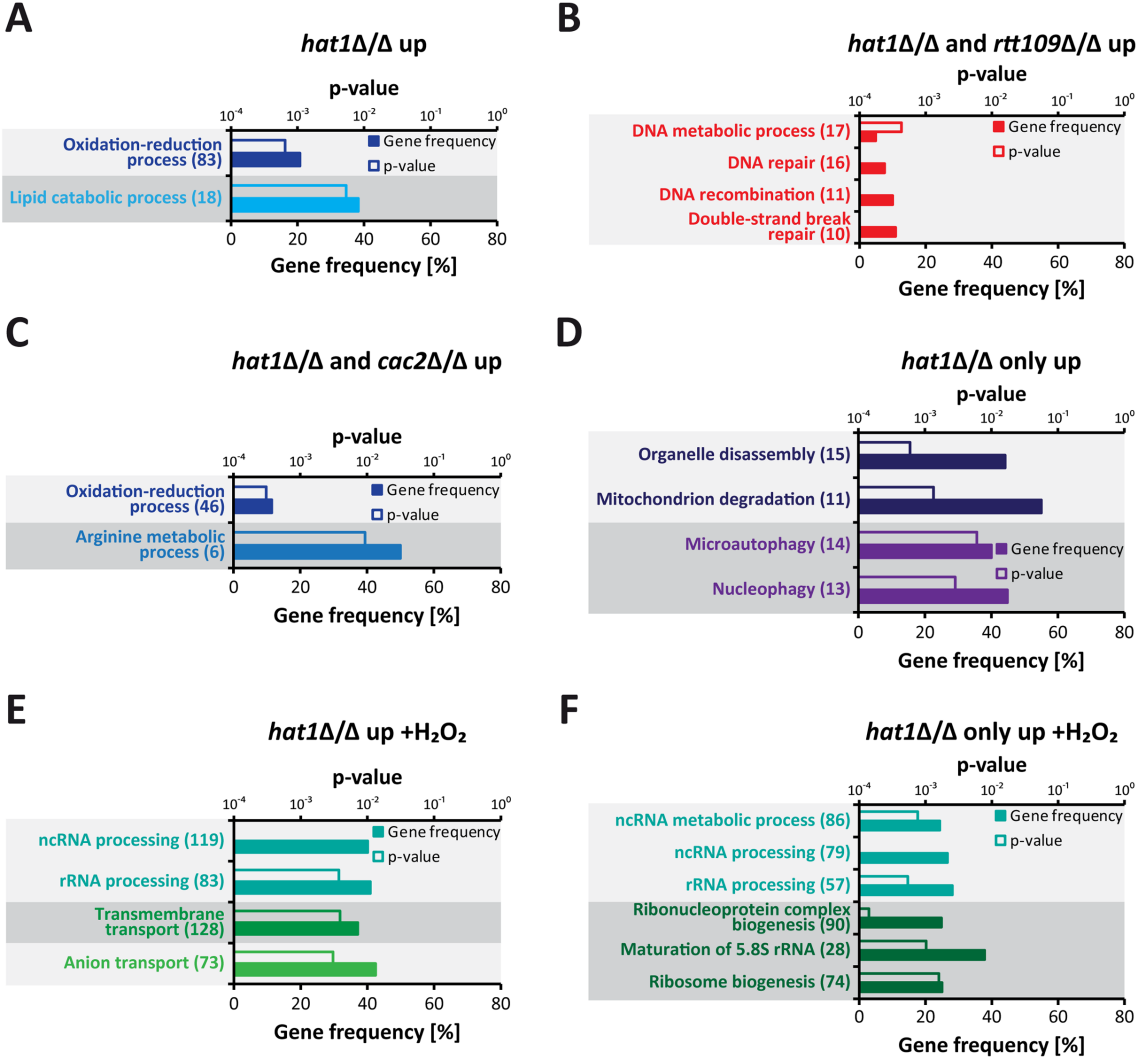
Specific functional gene groups are upregulated in cells lacking Hat1. (A) GO terms enriched among 2-fold significantly upregulated genes in logarithmically growing *hat1*Δ/Δ cells are shown. (B) The plot shows GO terms found within genes significantly upregulated in the *hat1*Δ/Δ and *rtt109*Δ/Δ strains only. (C) GO terms enriched within genes significantly upregulated in the *hat1*Δ/Δ and *cac2*Δ/Δ strains only. (D) The panel shows GO terms found among genes significantly upregulated in the *hat1*Δ/Δ mutant only and not in the *rtt109*Δ/Δ and the *cac2*Δ/Δ strains. (E) GO terms enriched among significantly upregulated genes in *hat1*Δ/Δ cells after treatment with H_2_O_2_ are shown. (F) The plot shows GO terms found within genes significantly upregulated in the *hat1*Δ/Δ strain only and not in the *rtt109*Δ/Δ and the *cac2*Δ/Δ strains upon H_2_O_2_ treatment. (A-F) The corresponding p-values for the enrichment (empty bars) and the percentage of genes changed within the GO group (filled bars) are presented. The absolute number of regulated genes within a GO group is presented in brackets. Groups containing identical genes are depicted in the same color. Significantly regulated genes were defined by a p-value <0.05.

To further characterize genes upregulated upon loss of Hat1, we performed a GO term enrichment analysis. Without H_2_O_2_ treatment, at least 2-fold upregulated genes in the *hat1*Δ/Δ mutant were strongly enriched for genes involved in lipid catabolic processes and oxidation-reduction processes (Fig 5A). Interestingly, the latter group includes genes encoding for proteins with functions in oxidative stress tolerance like the catalase (*CAT1*), superoxide dismutases (*SOD3-6*) and a thiol peroxidase (orf19.87) [26,54–56]. Preliminary proteomics data also identified this group of proteins as being upregulated in the *hat1*Δ/Δ mutant (S2C Fig). Of note, we failed to observe enrichment for DNA damage response genes most likely due to the fact that Hat1 is involved in different processes in *C*. *albicans*. This leads to a high number of differentially expressed genes in the mutant and could hamper detection of enriched GO groups. Therefore, we analyzed subsets of differentially expressed genes based on their expression in the three mutants. As expected, due to their DNA damage phenotype, genes significantly upregulated only in the *hat1*Δ/Δ and the *rtt109*Δ/Δ mutants were strongly enriched for DNA damage repair genes (Fig 5B). The specific overlap of regulated genes between *hat1*Δ/Δ and *cac2*Δ/Δ was still enriched for genes involved in oxidation-reduction processes and arginine metabolism, implying that Hat1 and Cac2 might be involved in the same processes (Fig 5C). Finally, we identified genes involved in mitochondrial degradation and microautophagy which were significantly upregulated only in the *hat1*Δ/Δ mutant (Fig 5D).

Next, we analyzed genes with differential expression levels in the mutants relative to the wild-type upon H_2_O_2_ treatment. The whole group of genes significantly upregulated in the *hat1*Δ/Δ mutant was enriched for genes involved in rRNA processing and transport reactions (Fig 5E). We failed to detect any gene sets enriched specifically in the overlaps between the *hat1*Δ/Δ and the *rtt109*Δ/Δ or the *hat1*Δ/Δ and *cac2*Δ/Δ strains. However, we observed a strong enrichment of genes involved in non-coding RNA (ncRNA)/rRNA processing and ribosome biogenesis as upregulated specifically in the *hat1*Δ/Δ mutant (Fig 5F).

In summary, loss of Hat1 mainly leads to increased expression of genes belonging to different functional groups, which is in accordance with Hat1 being involved in different processes in *C*. *albicans*. Furthermore, distinct functional groups affected by the loss of Hat1 are also upregulated in either the *rtt109*Δ/Δ or the *cac2*Δ/Δ strain indicating that Hat1 might function in specific processes together with Rtt109 or Cac2.

### Deletion of *HAT1* accelerates induction of oxidative stress genes

Transcriptional profiling of *hat1*Δ/Δ and *cac2*Δ/Δ cells revealed the upregulation of various gene sets encoding for proteins involved in the response to oxidative stress. Both mutants displayed elevated transcriptional levels of *CAT1* encoding catalase, which is responsible for the decay of H_2_O_2_ [54]. Of note, RT-qPCR analysis confirmed derepression of *CAT1* in *hat1*Δ/Δ and *cac2*Δ/Δ cells in the absence of H_2_O_2_. However, as expected from the RNA-Seq data, *CAT1* derepression was also observed for the *rtt109*Δ/Δ strain, which is not resistant to H_2_O_2_ (S2D Fig). Thus, this moderate derepression of *CAT1* is most likely due to a general stress response in these deletion mutants.

**Fig 6:**
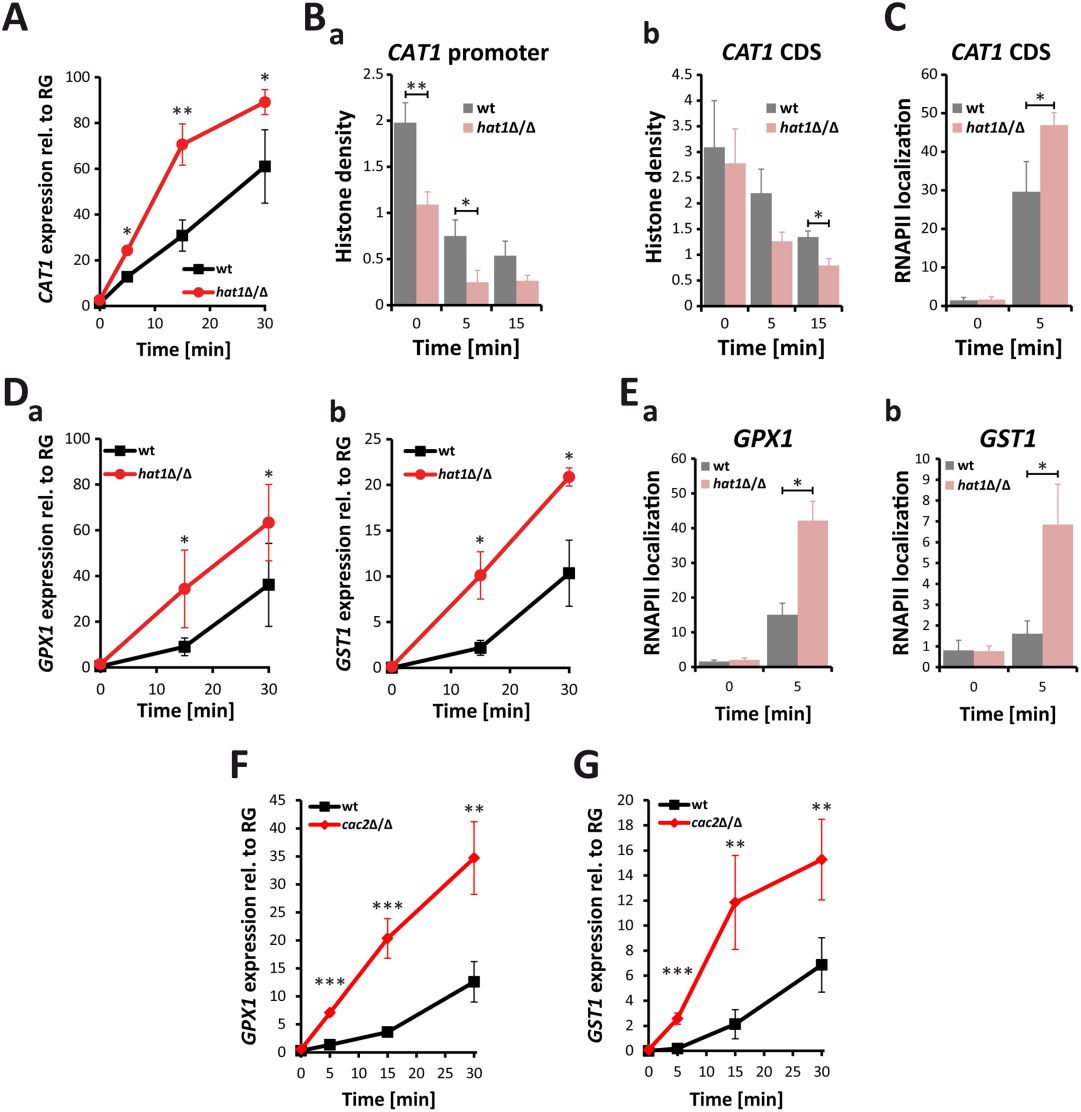
Lack of Hat1 accelerates induction of oxidative stress genes. (A) Catalase induction rate is strongly increased in *hat1*Δ/Δ cells. *CAT1* expression levels were measured by RT-qPCR after induction with 1.6 mM H_2_O_2_ at the indicated time points. Transcript levels were normalized to the expression level of the reference gene (RG) *PAT1*. Data are shown as mean + SD from 3 independent experiments. (B) Histone density at the *CAT1* locus is reduced in cells lacking Hat1. Histone H3 occupancy was determined by ChIP at the *CAT1* promoter region (a) and the CDS (b). (C) Loss of Hat1 leads to increased RNAPII recruitment at the *CAT1* locus. RNAPII levels were determined by ChIP at the *CAT1* CDS. (D) Induction rate of glutathione-utilizing enzymes is increased in hat1Δ/Δ cells. *GPX1* (a) and *GST1* (b) expression levels were determined by RT-qPCR at the indicated time points. Experiment was performed as described in (A). (E) Lack of Hat1 leads to increased RNAPII recruitment at the *GPX1* and *GST1* loci. RNAPII levels were determined by ChIP at the *GPX1* (a) and *GST1* (b) genes. (F+G) Loss of Cac2 increases the induction rate of both *GPX1* and *GST1* following H_2_O_2_ treatment. Experimental conditions were used as described in (A).

Therefore, we investigated *CAT1* induction upon treatment with H_2_O_2_. Interestingly, lack of Hat1 as well as Cac2 caused increased *CAT1* expression, indicating that these two proteins negatively regulate the induction kinetics of *CAT1* (S2E Fig). However, induction levels in the *rtt109*Δ/Δ control strain were similar to the wild-type (S2E Fig). Since lack of Hat1 affected induction levels of *CAT1*, we investigated the induction kinetics of this gene in detail. Thus, we determined transcript levels upon treatment with H_2_O_2_ over time. Interestingly, lack of Hat1 resulted in a significantly faster induction of the *CAT1* gene when compared to the wild-type (Fig 6A). Since Hat1 is involved in the deposition of histones into chromatin, we determined the effect of *HAT1* deletion on the histone density at the *CAT1* locus by chromatin immunoprecipitation (ChIP) using antibodies against histone H3. Without treatment, the *hat1*Δ/Δ mutant showed a reduced histone density at the *CAT1* promoter (Fig 6Ba). There was no difference in the occupancy in the *CAT1* coding sequence (CDS) between the mutant and the wild-type (Fig 6Bb). Interestingly, however, treatment with H_2_O_2_ decreased histone density at the promoter as well as in the CDS significantly faster in *hat1*Δ/Δ cells when compared to the wild-type (Fig 6B). This explains the increased induction rate in the mutant, since nucleosomes represent a physical barrier for RNA polymerase II (RNAPII) and reduced nucleosome density can facilitate transcription [6,35,57]. Thus, lower histone density in the *hat1*Δ/Δ mutant could promote higher RNAPII processivity leading to increased mRNA production. Alternatively, elevated mRNA levels could also be due to increased RNAPII recruitment, which could be facilitated by the reduction in nucleosome density at the promoter. To clarify whether elevated mRNA levels are the consequence of increased RNAPII recruitment, we determined RNAPII levels at the *CAT1* gene using ChIP. Interestingly, we detected increased recruitment of RNAPII to the *CAT1* CDS, thus explaining the elevated mRNA levels (Fig 6C).

Notably, the oxidative stress response set upregulated in the *hat1*Δ/Δ mutant also comprised several glutathione-utilizing enzymes. Thus, we determined the induction kinetics for two of these genes upon treatment with H_2_O_2_. Similar to *CAT1*, we detected a faster induction of *GPX1* (orf19.86) encoding a glutathione peroxidase (GPx) as well as of *GST1* (orf19.3121), encoding a glutathione S-transferase (Fig 6D). In addition, we also detected increased RNAPII levels at both genes upon treatment with H_2_O_2_ (Fig 6E). Notably, we observed similar hyperinduction of *GPX1* and *GST1* upon H_2_O_2_ treatment for the *cac2*Δ/Δ mutant as well (Fig 6F and 6G).

**Fig 7:**
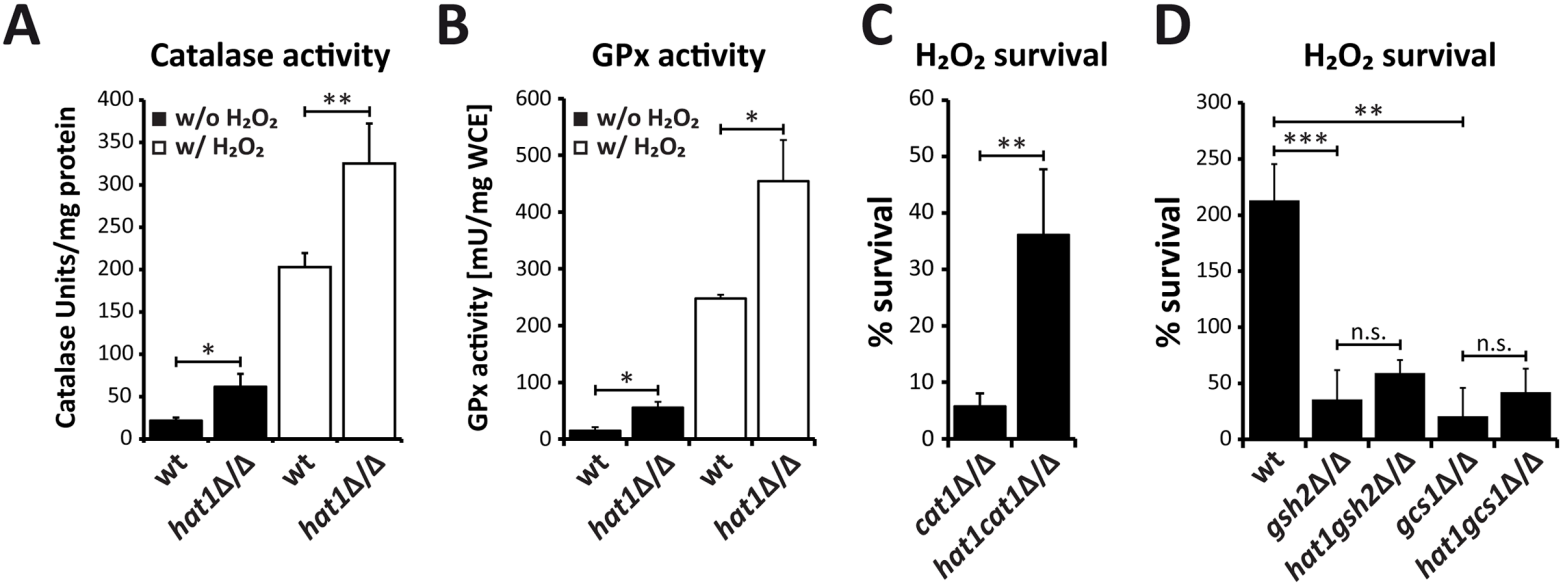
Loss of Hat1 raises antioxidant enzyme activity and glutathione-mediated H_2_O_2_ resistance. (A) Faster *CAT1* induction increases catalase activity in *hat1*Δ/Δ cells. Catalase activity was determined in whole cell extracts isolated from cells before and after H_2_O_2_ treatment. Data are shown as mean + SD from three independent experiments. (B) Loss of Hat1 leads to increased glutathione peroxidase activity. GPx activity was determined in whole cell extracts isolated from cells before and after H_2_O_2_ treatment. Data are shown as mean + SD from two independent experiments. (C) Lack of *CAT1* does not abolish Hat1-mediated H_2_O_2_ resistance. Cells of the indicated strains were treated with 1 mM H_2_O_2_ for 2 hours, plated and colonies counted after 3 days of incubation on YPD plates at 30°C to determine viability. Data are shown as mean + SD from three independent experiments. (D) Depletion of glutathione biosynthesis abolishes Hat1-mediated H_2_O_2_ resistance. Cells of the indicated strains were treated with H_2_O_2_ for 2 hours and plated on YPD plates containing glutathione. Colonies were counted to determine viability after growth for 3 days at 30°C. Data are shown as mean + SD from three independent experiments. (A-D) n.s.: not significant, *P<0.05, **P<0.01 and ***P<0.001 relative to the corresponding control (Student's t-test).

Furthermore, we tested whether the increase in transcription of oxidative stress genes in the *hat1*Δ/Δ mutant is paralleled by elevated activity of the corresponding enzymes. Therefore, we prepared whole cell extracts from cells before and after 60 min exposure to H_2_O_2_ and determined catalase activity as well as GPx activity spectrophotometrically. We detected elevated catalase activity in the *hat1*Δ/Δ mutant already under non-inducing conditions, albeit as expected at a low level (Fig 7A). However, H_2_O_2_ treatment of *hat1*Δ/Δ cells dramatically increased catalase activity when compared to the wild-type, indicating that the mutant is more efficient in degrading hydrogen peroxide (Fig 7A). Likewise, the GPx activity assay detected some increase already under non-inducing conditions (Fig 7B), but H_2_O_2_ treatment of the *hat1*Δ/Δ mutant significantly increased GPx activity (Fig 7B). These data demonstrate that the increased induction rate of oxidative stress genes in the *hat1*Δ/Δ mutant increases the activities of the enzymes encoded by the target genes.

Furthermore, to determine whether catalase hyperinduction is the main reason for the increased resistance of the *hat1*Δ/Δ mutant, we deleted the *CAT1* gene in wild-type and *hat1*Δ/Δ cells and quantified their H_2_O_2_ susceptibilities. As expected, *cat1*Δ/Δ cells were highly sensitive to H_2_O_2_ showing only 6% survival after treatment with a low H_2_O_2_ concentration of 1 mM (Fig 7C). However, the double deletion strain maintained increased H_2_O_2_ resistance when compared to the *cat1*Δ/Δ single mutant indicating that increased catalase expression is not the main reason for the hydrogen peroxide resistance of the *hat1*Δ/Δ strain (Fig 7C). To determine if increased induction of glutathione-utilizing enzymes is causing the increased H_2_O_2_ resistance, we genetically depleted cells for glutathione by removing the *GSH2* and *GCS1* glutathione biosynthesis genes in wild-type and *hat1*Δ/Δ backgrounds. The first step in the synthesis of glutathione is catalyzed by the gamma-glutamylcysteine synthetase (Gcs1), followed by the second step carried out by the glutathione synthase (Gsh2). Deletion of one of these genes disrupts glutathione biosynthesis in *C*. *albicans* leading to glutathione auxotrophy and to increase oxidative stress sensitivity [58,59]. To determine the sensitivity to H_2_O_2_ of wild-type and *hat1*Δ/Δ cells upon deletion of *GSH2* or *GCS1*, the strains were grown in the absence of glutathione and treated with H_2_O_2_ for 2h. As expected, lack of Gsh2 as well as Gcs1 strongly increased the sensitivity to H_2_O_2_ (Fig 7D). Strikingly, however, the absence of Hat1 did not significantly change H_2_O_2_ resistance in the absence of Gsh2 or Gcs1 (Fig 7D). Thus, the data strongly suggest that H_2_O_2_ resistance caused by deletion of Hat1 is mainly mediated via the glutathione system.

### Hat1 regulates ROS detoxification and neutrophil survival

**Fig 8:**
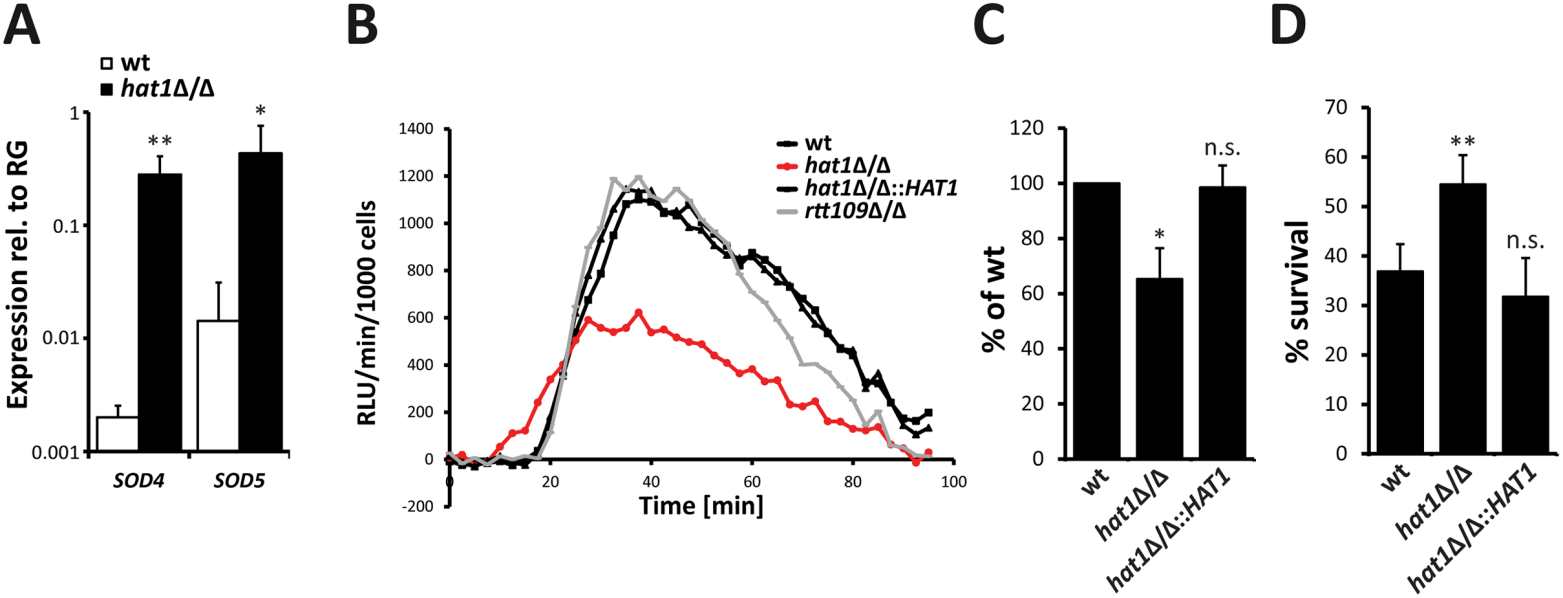
Higher ROS detoxification capacity of *hat1*Δ/Δ cells causes resistance to neutrophil killing. (A) Superoxide dismutases Sod4 and Sod5 are induced in *hat1*Δ/Δ cells. Expression levels of *SOD4* and *SOD5* in logarithmically growing cells were detected by RT-qPCR. Transcript levels were normalized to the expression level of the reference gene (RG) *RIP1*. Data are shown as mean + SD from 3 independent experiments. (B) Infection of macrophages with *hat1*Δ/Δ cells causes reduced ROS accumulation. ROS levels were determined by measuring luminol-dependent chemiluminescence [relative luciferase units (RLU) min^-1^ per 1000 immune cells] in 2.5 min intervals during interaction of the indicated *C*. *albicans* strains with bone marrow-derived murine macrophages (BMDMs). One representative experiment is shown. Data were reproduced in three independent experiments. (C) Quantification of total ROS release upon interaction with BMDMs. Experiment was performed as described in (B). The area under the curve within 90 min of interaction was calculated. Data are shown as mean + SD from three independent experiments. (D) Cells lacking Hat1 show increased survival to neutrophil killing. Survival of *C*. *albicans* cells upon one hour interaction with murine bone marrow neutrophils was determined by plating and CFU counting. Data are shown as mean + SD from three independent experiments. (A-D) *P<0.05, **P<0.01 relative to the wild-type (Student's t-test).

The absence of Hat1 and Cac2 leads to upregulation of oxidative stress genes and resistance to hydrogen peroxide. ROS are produced by immune cells to kill *C*. *albicans* during the infection process. The fungus counteracts this attack by upregulating ROS detoxifying enzymes of which the superoxide dismutases Sod5 and to some extend Sod4 were shown be essential for survival of *C*. *albicans* upon phagocytosis [26]. Interestingly, our RNA-Seq data not only revealed an upregulation of the catalase and glutathione-utilizing enzymes, but also markedly increased expression of genes encoding the superoxide dismutases Sod4 and Sod5 in cells lacking Hat1. The significant induction of *SOD4* and *SOD5* in the *hat1*Δ/Δ strain was also confirmed by RT-qPCR analysis (Fig 8A).

Therefore, we asked if the deletion of *HAT1* influences the ability to detoxify and survive ROS released by immune cells. ROS production during interaction of *C*. *albicans* with bone marrow-derived murine macrophages and bone marrow neutrophils was determined by measuring luminol dependent chemiluminescence [26]. Interestingly, deletion of *HAT1* strongly reduced total ROS levels during interaction with macrophages (Fig 8B and 8C), as well as neutrophils (S3A Fig), when compared to the wild-type. Phagocytosis is required for ROS production by the NADPH oxidase [26]. To exclude that differences in phagocytosis of the pseudohyphal *hat1*Δ/Δ cells contribute to reduced ROS levels, we performed a phagocytosis assay. However, phagocytosis was similar for the *hat1*Δ/Δ strain or wild-type (S3B Fig). Furthermore, we also determined the NADPH oxidase activation in macrophages interacting with wild-type and *hat1*Δ/Δ cells by immunoblotting. However, we observed no difference in the phosphorylation levels of the NADPH oxidase subunit p40phox upon interaction with *hat1*Δ/Δ cells or wild-type cells (S3C Fig). Therefore, reduced ROS accumulation upon interaction with *hat1*Δ/Δ cells most likely results from increased ROS detoxification by the mutant. In addition, interaction of macrophages with *rtt109*Δ/Δ cells did not lead to significant changes in the ROS levels when compared to the wild-type (Fig 8B).

Finally, we determined the resistance of *hat1*Δ/Δ cells to killing by bone marrow neutrophils. Strikingly, lack of Hat1 clearly increased the survival rate upon interaction with the immune cells. Furthermore, reintegration of *HAT1* fully restored the wild-type sensitivity (Fig 8D). These data strongly suggest that genetic removal of *HAT1* strongly promotes increased survival to neutrophil attack due to the impaired ROS accumulation.

### Cells lacking Hat1 show reduced virulence but persist in mouse kidneys

**Fig 9:**
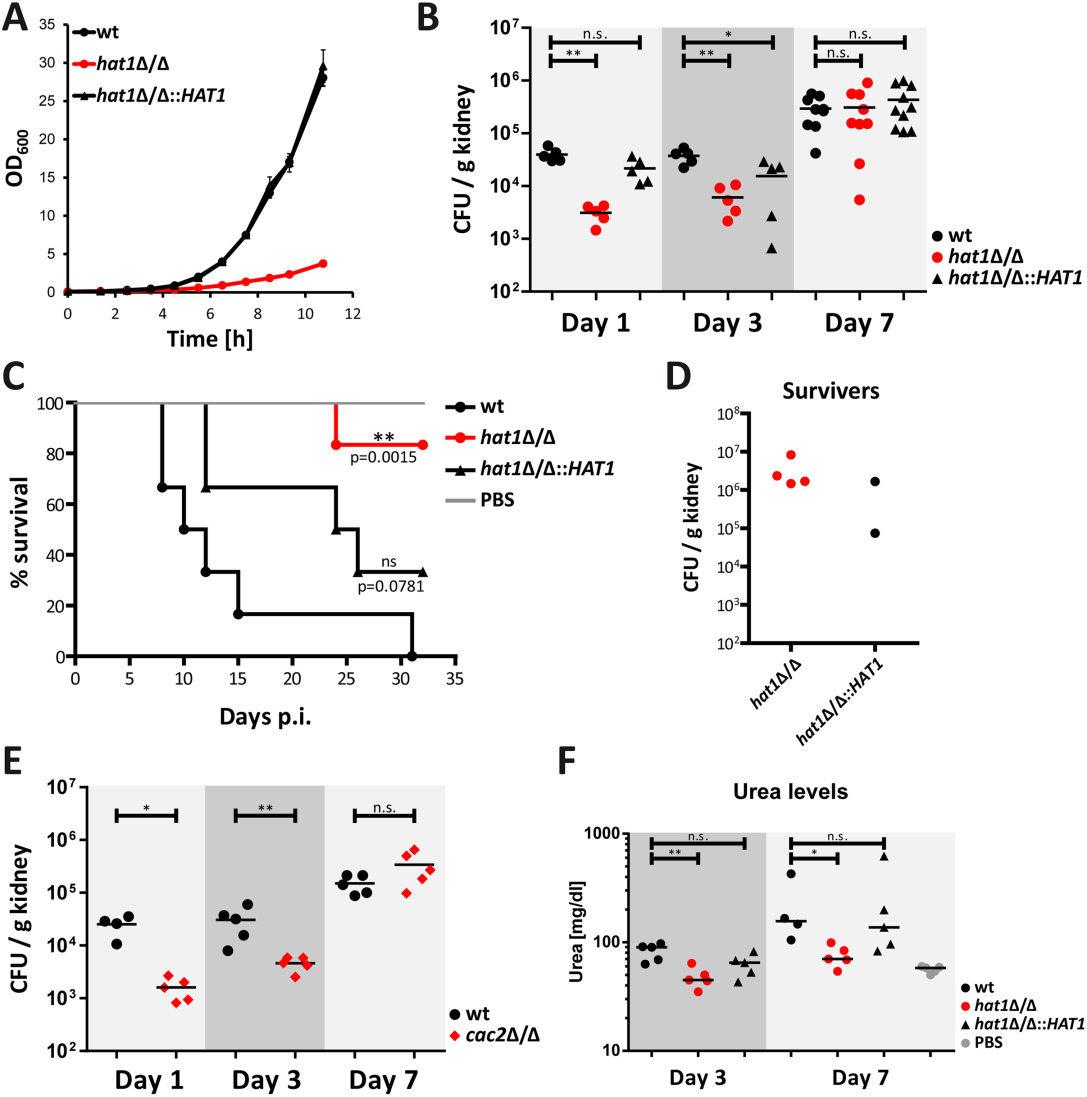
Cells lacking Hat1 show reduced virulence but persist in mouse kidneys. (A) Reduced growth rate of the *hat1*Δ/Δ strain was determined by measuring the OD_600_ of cells growing in YPD at 30°C. (B) Cells lacking Hat1 are not cleared efficiently from kidneys. At the indicated time points, fungal burdens in kidneys of mice infected with *C*. *albicans* strains were determined and expressed as CFUs per gram kidney. Groups of 5–10 mice were analyzed at each time point and statistical significance was determined using the non-parametric Mann-Whitney-test. n.s.: not significant, *P<0.05 and **P<0.01 relative to the corresponding wild-type. (C) *hat1*Δ/Δ cells are defective in killing the host. Survival of mice infected with the indicated strains was monitored over 32 days post infection (p.i.). The data are presented as Kaplan-Meier survival curves. Groups of 6 mice were infected per *C*. *albicans* strain. Statistical significance was determined using the Log-rank test. ns: not significant; (D) Fungal burdens in kidneys of surviving mice from panel C were determined and expressed as CFUs per gram organ. One mouse infected with the *hat1*Δ/Δ strain was able to clear *Candida*. (E) The *cac2*Δ/Δ strain is not cleared efficiently from kidneys. Experiment was performed as described in (B). Groups of 4–5 mice were analyzed at each time point. (F) Infection with *hat1*Δ/Δ cells causes reduced kidney damage. Urea levels were determined in sera of infected mice at day 3 and 7 post infection. n.s.: not significant, *P<0.05, **P<0.01 relative to the wild-type (Student's t-test).

Deletion of *HAT1* causes reduced growth rate *in vitro* with morphological defects even in complete media (Fig 9A) [10], which has been shown to reduce virulence of several *C*. *albicans* mutants [28,30,60–63]. However, cells lacking Hat1 are also more resistant to killing by immune cells (Fig 8D). Therefore, we wanted to test how these seemingly opposing phenotypes caused by the deletion of *HAT1* affect virulence of *C*. *albicans*. We used a mouse model of systemic candidiasis. Infection was performed via the tail vein and fungal burdens in kidneys were followed at day 1, 3 and 7. Interestingly, after 24 hours mice infected with the *hat1*Δ/Δ mutant showed significantly reduced CFUs in the kidneys when compared to the wild-type or the restored strain (Fig 9B). However, the fungal burden of the mutant increased until day 7 after infection reaching the levels of the wild-type and the reintegrant (Fig 9B). Thus, cells lacking Hat1 are not efficiently cleared from infected mice as they are able to compensate the *in vitro* growth defect *in vivo*. To further investigate the virulence properties of the *hat1*Δ/Δ strain, we determined the survival rate of infected mice. Interestingly, 15 days post infection the majority of wild-type infected mice had died, whereas all of the mice infected with the *hat1*Δ/Δ strain were still alive (Fig 9C). Even after 32 days, only one mouse infected with the *hat1*Δ/Δ mutant had died. Of note, mice infected with the reintegrant showed an intermediate survival rate, which was however not significant when compared to the wild-type strain (Fig 9C). Although the majority of mice survived the infection with cells lacking Hat1, mutant cells were not cleared from the kidneys in 4 out of 5 individuals (Fig 9D). Instead, the fungal burden stayed high until the end of the experiment. Furthermore, two mice survived infection with the restored strain and for both *Candida* was not cleared (Fig 9D). Thus, again the revertant strain showed an intermediate phenotype most likely due to haploinsufficiency.

We also determined the effect of deleting *CAC2 in vivo* using the same murine model of systemic infection as for the *hat1*Δ/Δ mutant. Interestingly enough, *cac2*Δ/Δ cells showed reduced fungal burdens at day 1 and 3 after infection. However, similar to the *hat1*Δ/Δ mutant, the strain lacking Cac2 was also protected from clearance by the immune system, since CFUs in kidneys increased during the course of the experiment (Fig 9E). In addition, body weight measurements of infected mice revealed a striking difference between the wild-type and *hat1*Δ/Δ as well as the *cac2*Δ/Δ cells. As expected, mice infected with the wild-type rapidly lost body weight after infection. However, upon infection with the *hat1*Δ/Δ or the *cac2*Δ/Δ strain, weight loss was clearly reduced, suggesting a reduced burden to the immune system or increased tolerance to the infecting fungal strain (S4A and S4B Fig).

Nonetheless, although showing increased persistence in the host, deletion of *HAT1* strongly attenuates virulence in a mouse model of systemic candidiasis. Even though fungal burdens are comparable between the *hat1*Δ/Δ mutant and the wild-type 7 days post infection, the mutant is strongly attenuated in killing the host. Since kidney failure is the primary cause of death in this particular mouse infection model [64,65] we determined the degree of kidney injury or function in infected mice by measuring serum urea levels. As expected due to the lower fungal burden, mice infected with the *hat1*Δ/Δ mutant showed reduced urea levels at day 3. The reintegrant again yielded in an intermediate phenotype (Fig 9F). However, at day 7 lack of Hat1 still yielded in lower urea levels when compared to the wild-type (Fig 9F). Thus, lack of Hat1 seems to cause reduced kidney injury even at comparable fungal burdens. In summary, our data suggest that the *hat1*Δ/Δ strain is able to compensate its *in vitro* growth defect *in vivo* and persist in the host. However, cells lacking Hat1 are severely compromised in killing the host.

## Discussion

### Regulation of oxidative stress genes via chromatin assembly

In this work, we provide compelling evidence for a novel function of the eukaryotic histone acetyltransferase Hat1 in the pathogenic fungus *C*. *albicans*. Hat1 is required for regulating oxidative stress response, antifungal drug tolerance as well as virulence. We show that Hat1 functions in distinct chromatin assembly pathways acting in concert with well-known histone chaperones CAF-1 and HIR. Hence, in addition to an evolutionary conserved role of Hat1 in DNA damage repair [10,41,66,67] our data demonstrate a novel function for Hat1 in regulating the response to oxidative stress and azole treatment. Furthermore, certain resistance phenotypes as well as gene sets misregulated upon loss of Hat1 are also affected by distinct histone chaperones operating in independent chromatin assembly pathways. Thus, our data suggest that the Hat1 histone acetyltransferase has a central function in mediating the flux of histones necessary for nucleosome remodeling in *C*. *albicans* thereby affecting various cellular processes, including fungal virulence and persistence in the host.

Interestingly, the function in the regulation of the ROS response seems restricted to *C*. *albicans* and related fungal CTG clade members. This has not been reported in other organisms so far. This function of Hat1 might have developed only recently, providing an explanation why this is only observed in a small number of fungal species. Of note, Rtt109 from non-pathogenic *S*. *cerevisiae* negatively regulates catalase expression in response to H_2_O_2_ together with Asf1 via histone deposition, whereby lack of Asf1 or Rtt109 increases RNAPII recruitment [33]. Likewise, we detect increased RNAPII levels at several oxidative stress genes immediately after induction in the *hat1*Δ/Δ mutant (Fig 6). While the *S*. *cerevisiae rtt109*Δ mutant displays slightly increased H_2_O_2_ resistance (S5 Fig), the *C*. *albicans RTT109* deletion fails to effect catalase induction or peroxide resistance (S1C and S2E Figs). Thus, it is very tempting to speculate that Hat1 and Rtt109 are involved in the regulation of distinct gene sets in different species. Noteworthy, Asf1 interacts with Hat1 in *S*. *cerevisiae* [68]. Whether Hat1 regulates oxidative stress response in *C*. *albicans* also via Asf1 remains unclear, since Asf1 is an essential gene in *C*. *albicans*. Asf1 is also essential in *S*. *pombe* and *Drosophila melanogaster* [69,70], implying increased functional redundancy in histone chaperone functions in *S*. *cerevisiae* when compared to *C*. *albicans* and other species.

Of particular interest is that Spt6 negatively regulates catalase expression in *S*. *cerevisiae* [33]. The observed H_2_O_2_ resistance phenotype of the *C*. *albicans spt6*Δ/Δ mutant might indicate conservation of this function in both species. Although interesting, further experiments are needed to determine the molecular mechanism(s) of oxidative stress regulation in cells lacking Spt6.

We show here that genetic removal *HAT1* accelerates the induction kinetics of oxidative stress genes, including catalase as well as other genes encoding glutathione-utilizing enzymes. However, the lack of catalase in a *hat1*Δ/Δ background does not lower the H_2_O_2_ sensitivity to the level of the *cat1*Δ/Δ single knock-out (Fig 7C). On the other hand, the absence of Hat1 combined with defects in glutathione biosynthesis does not significantly increase H_2_O_2_ tolerance (Fig 7D). Therefore, the H_2_O_2_ resistance phenotype of the *hat1*Δ/Δ deletion strain is likely to be due to the upregulation of glutathione-utilizing enzymes, although a contribution of Cat1 cannot be fully excluded at this point.

### Repression of genes via distinct chromatin assembly pathways

Interestingly, novel functions for the CAF-1 and HIR histone chaperone complexes in the regulation of white-opaque switching were reported for *C*. *albicans* [71]. The CAF-1 and HIR chaperone complexes are essential for histone deposition via distinct pathways. While the HIR complex functions in replication-independent chromatin assembly, CAF-1 is thought to mediate replication-coupled histone deposition [21,44,72].

To the best of our knowledge, no reports exist suggesting a role for Hat1 acting through the HIR complex to modulate antifungal drug resistance in any other eukaryote. Of note, this Hat1 function may have been conserved in some fungal species, since elevated azole tolerance is at least observed in *S*. *pombe* lacking the Hat1 orthologue. Of note, *HAT1* is indeed repressed in *C*. *albicans* cells treated with itraconazole, implying a function for Hat1 in the regulation of azole susceptibility [73]. Furthermore, our RNA-Seq data demonstrate a downregulation of ergosterol biosynthesis genes in the *hat1*Δ/Δ strain, including *ERG3* whose lack confers pronounced azole resistance [74]. Thus, a similar mechanism may explain the reduced susceptibility of *hat1*Δ/Δ and *hir1*Δ/Δ strains. Another mechanism mediating the increased azole tolerance could be the upregulation of multidrug transporters [75,76]. The two major ATP-binding cassette transporters responsible for azole resistance in *C*. *albicans* are Cdr1 and Cdr2 [77–80]. Whereas only *CDR2* was slightly upregulated in *hat1*Δ/Δ cells, the major facilitator superfamily transporter Mdr1, which is also induced upon oxidative stress and confers azole resistance [77,81], is significantly upregulated in the *hat1*Δ/Δ mutant upon treatment with H_2_O_2_ (S1 Table). However, the exact mechanism by which Hat1 and Hir1 regulate azole tolerance remains to be determined in future studies.

Although, the CAF-1 complex can assemble histones in a replication-coupled manner, it can also influence the rate of replication-independent histone incorporation and transcription [22,82,83]. Therefore, NuB4 and CAF-1 might act in concert to regulate specific target genes such as oxidative stress response sets in *C*. *albicans*. We propose that they do so by facilitating histone incorporation concomitant with transcription, thereby increasing the histone density. Indeed, we observe a large overlap in differentially expressed gene sets in *hat1*Δ/Δ and the *cac2*Δ/Δ mutants, the latter lacking a subunit of CAF-1 (Fig 4D and 4E). Unfortunately, we were not able to obtain a *hat1*Δ/Δ *cac2*Δ/Δ double deletion strain for epistasis analysis. Thus, we cannot completely rule out the possibility that Hat1 and Cac2 regulate H_2_O_2_ resistance independently. One explanation for a possible synthetic lethality of *hat1*Δ/Δ *cac2*Δ/Δ double mutants may be that both are associated with essential DNA replication [21,84,85].

The genome-wide transcriptional RNA-Seq profiling of logarithmically growing *hat1*Δ/Δ cells mainly revealed upregulation of genes, suggesting repressive functions of Hat1. Notably, distinct GO terms are enriched in the *hat1*Δ/Δ mutant alone or in overlaps with other mutants. These data suggest specific functions for Hat1 in different processes, including DNA damage repair, arginine biosynthesis and mitochondrial degradation (Fig 5). Interestingly, Rtt109 inhibits arginine biosynthesis genes in *S*. *cerevisiae* together with Asf1 under repressing conditions [86]. By contrast, this gene set is derepressed in *C*. *albicans hat1*Δ/Δ and *cac2*Δ/Δ, but not in *rtt109*Δ/Δ cells (Fig 5B and 5C). Thus, we believe that Hat1/Cac2 have taken over this function from Rtt109 in *C*. *albicans*. Interestingly enough, mitochondrial dysfunction has been linked to azole and oxidative stress sensitivity in *C*. *albicans*, as well as in other fungal species [87–89]. However, depletion of Fzo1, which is involved in mitochondrial fusion, and Goa1, a protein localizing to mitochondria upon oxidant treatment [87,88], decreases azole and oxidative stress tolerance. Thus, although genes involved in mitochondrion degradation are upregulated in *hat1*Δ/Δ cells, the loss of mitochondrial function is unlikely to explain the increase in stress resistance.

Interestingly, upon H_2_O_2_ treatment we observed increased expression of genes involved in ncRNA/rRNA processing specifically in the *hat1*Δ/Δ mutant (Fig 5E and 5F). However, this group of genes is repressed in the wild-type upon H_2_O_2_ stress. Only 4 out of a total of 119 *hat1*Δ/Δ upregulated genes in this group were less than 2-fold repressed in the wild-type. Thus, lack of Hat1 seems to cause a defect in repression of genes involved in ncRNA/rRNA processing and ribosome biogenesis. Although a faster induction kinetics of genes connected to H_2_O_2_ resistance occurs upon loss of Hat1 (Fig 6A and 6D), many oxidative stress-regulated genes appear not significantly modulated in the RNA-Seq dataset making GO term enrichment for this group futile. A possible explanation is that many stress conditions in fungi, including oxidative stress or osmostress, follow a stress-specific and often transient regulation [90], which may result in difficulties choosing the correct timing for collecting data points in *hat1*Δ/Δ and wild-type cells. Nevertheless, we speculate that the Hat1-mediated oxidative stress regulation may relate to host infection conditions, where severe oxidative stress is a major immune defense during phagocytosis of fungal pathogens [26,91].

### Inactivation of Hat1 compromises virulence in *C*. *albicans*

Although infection of macrophages or neutrophils by the *hat1*Δ/Δ mutant strongly reduces total ROS accumulation, we observed faster accumulation of ROS in the beginning of the interaction (Fig 8B and S3A Fig). Differences in ROS production cannot be simply explained by the pseudohyphal morphology of cells lacking Hat1, since the *rtt109*Δ/Δ strain has the same morphological defect [30] yet does not show any differences in ROS accumulation (Fig 8B). Thus, it may specifically result from Hat1-mediated gene regulation and from upregulated factors triggering ROS production. Interestingly enough, a mouse model of virulence yields compelling data showing that *hat1*Δ/Δ cells display strongly attenuated virulence. Of note, infected mice show significantly reduced organ burdens of *hat1*Δ/Δ cells immediately after infection, but reach comparable levels of fungal burdens between day 3 and day 7 post infection (Fig 9B). This implies a faster growth *in vivo*, which may be explained by the reduced susceptibility to neutrophil-mediated killing, essentially providing a “fitness” advantage of *hat1*Δ/Δ *in vivo*. Moreover, cells lacking Hat1 cause less damage to host organs and therefore enable better mouse survival. Reduced urea levels in sera of infected mice support this notion (Fig 9F). In line with this hypothesis is also the fact that *hat1*Δ/Δ cells degrade ROS more efficiently, protecting the *hat1*Δ/Δ mutant from clearance by the immune surveillance. This may also result in reduced tissue damage, since reactive oxygen species originating from innate immune response are harming both the pathogen and the host due to increasing inflammation [92,93]. Indeed, a downmodulation of the inflammatory immune response can be beneficial for the host during invasive *Candida albicans* infections [65,94]. Finally, ROS is also known as a signaling molecule during infections and reduced levels upon challenge with the *hat1*Δ/Δ mutant might impact the immune response [95].

Furthermore, compelling evidence exists suggesting that the *hat1*Δ/Δ virulence phenotype is not just due to its role in DNA damage repair. Deletion of *RAD52* or *RTT109* also causes pseudohyphal cell morphology and strong virulence defects in mouse models of systemic candidiasis [28,30,96]. However, the *rtt109*Δ/Δ mutant is efficiently cleared from kidneys of infected mice 3 days post infection [30]. Likewise, kidney fungal burdens of *rad52*Δ/Δ cells decline over 3 days post infection even with an inoculum concentration ten times higher than in our setup [28]. By sharp contrast, the *hat1*Δ/Δ fungal burdens in kidneys increase, reaching wild-type levels 7 days post infection. Nonetheless, despite increased growth *in vivo*, removal of Hat1 renders cells unable to kill the host efficiently over 32 days, consistent with a persistent but avirulent invasive infection (Fig 9C).

### Evolution of specialized functions for chromatin assembly pathways in *C*. *albicans*

Our data unequivocally show that loss of Hat1, Cac2 and Hir1 can be beneficial for *C*. *albicans* in our experimental setup. However, the inability to efficiently repress genes under stress conditions might also be detrimental for the organism. Furthermore, loss of Hat1 and Cac2 also impairs DNA damage repair and thus promotes genome instability [10,71]. Thus, the resulting fitness loss due to the absence of these factors might be disadvantageous in the long run, despite a gain in oxidative resistance. Further experiments are required to identify additional factors functioning together with NuB4/CAF-1 and NuB4/HIR in the regulation of oxidative stress resistance or azole tolerance. This might also lead to the discovery of potential antifungal targets, which could be used to render cells more sensitive to ROS or azoles. Importantly, the data from the mouse model imply that inhibitors of HATs could have beneficial effects in clinical therapeutic settings but pose the risk of promoting persistent or latent infections.

Interestingly, we don’t observe comparable resistance phenotypes upon genetic removal of Hat1 in *S*. *cerevisiae*, *C*. *glabrata* and at least for the oxidative stress resistance also in *S*. *pombe* (Fig 3). Thus, in *C*. *albicans*, the NuB4 and CAF-1 complexes might have gained functions during coevolution with the human host, which appears restricted to the CTG clade. Hence, deletion of *HAT1* in *C*. *glabrata*, the second-most prevalent human fungal pathogen [24], does not result in similar phenotypes. Nonetheless, our results clearly demonstrate substantial differences in the functions of even highly conserved chromatin modification mechanisms between *C*. *albicans* and other fungi. Therefore, results obtained from classical model systems such as baker’s yeast often cannot simply be transferred to pathogenic fungi.

The regulation of virulence-associated traits by chromatin modification has been reported for several pathogens and it is now generally accepted that the chromatin status affects virulence [97,98]. Moreover, several reports suggest that chromatin modifiers are involved in the regulation of fungal virulence factors including antifungal drug tolerance [97,99–102]. Hence, modulation of chromatin function or chromatin-modifying pathways including nucleosome remodeling may be a common strategy during coevolution of microbial pathogens with the host to promote immune evasion.

## Materials and Methods

### Ethics statement

All animal experiments were evaluated by the ethics committee of the Medical University of Vienna and approved by the Federal Ministry for Science and Research, Vienna, Austria (GZ: BMWF- 68.20n5/231-II/3b/2011) adhering to European legislation for animal experimentation.

### Media, chemicals and growth conditions

Rich medium (YPD) and synthetic complete medium (SC) were prepared as previously described [103]. Minimal medium contained 8.38 g/l yeast nitrogen base (BD Biosciences), 6.25 g/l ammonium sulfate (Sigma Aldrich) and 2 g/l glucose (Sigma Aldrich). Fungal strains were routinely grown on YPD plates at 30°C. Hydrogen peroxide, tert-butyl hydroperoxide, Calcofluor White, Congo Red, cadmium chloride, sodium chloride, luminol and HRP Type VI were obtained from Sigma Aldrich. Voriconazole, Itraconazole and Amphotericin B were purchased from Discovery Fine Chemicals Ltd. DMEM and RPMI media were purchased from PAA.

### Plasmid and strain construction and genomic verification

A list of fungal strains, plasmids and primers used in this study is shown in Tables A, B and C in S1 Text, respectively. All *C*. *albicans* strains constructed in this work were derived from the clinical isolate SC5314 [104]. For deletion of *HAT1* in *C*. *parapsilosis* the clinical isolate GA1 was used [105]. Disruption of *HAT1* in *C*. *tropicalis* was done in the clinical isolate AKH2249. Deletion of *CAC2* and *HIR1* was done using a modified version of the SAT1 flipper method [106]. Briefly, the marker cassette was amplified using the pSFS3b plasmid and primers containing some 80bp homologous region to replace the whole coding sequence of the corresponding gene [10]. For deletion of *RTT106* two primer pairs were used to add the homologous regions in two sequential PCR steps. For deletion of *CAT1*, YEp352-SAT1-*CAT1*urdr was constructed by *in vivo* cloning in *S*. *cerevisiae* exactly as described previously [107]. Plasmids for deletion of *GCS1*, *GSH2*, *C*. *tropicalis HAT1* and *C*. *parapsilosis HAT1* were constructed by fusing ~500bp fragments upstream and downstream of the corresponding gene with a FRT-FLP-NAT1-FRT cassette derived from pSFS3b [10] and a fragment containing an ampicillin resistance cassette and an *E*. *coli* replication origin derived from YEp352 [108] via *in vivo* recombination in *E*. *coli* EL350 as described in [109]. Due to low transformation efficiency for *C*. *parapsilosis* and *C*. *tropicalis* a second set of deletion vectors was constructed in the same way to obtain the homozygous knock-outs. These vectors contained 300–350bp of the beginning and the end of the corresponding CDS for homologous recombination to avoid integration at the deleted allele. For construction of the *CAC2* reintegration plasmid the coding sequence plus ~500 up- and downstream was amplified and cloned into pSFS3b via *Kpn*I and *Apa*I. Transformation of *C*. *albicans* was done via electroporation [106]. Correct integration of the deletion cassette and loss of the corresponding gene were confirmed by colony PCR.

Colony PCR assays were used to verify correct genomic integration of deletion constructs. Briefly, a colony was resuspended in 25 μl H_2_O in a PCR tube and incubated at 95°C for 10 minutes. Cell debris was spun down briefly and 5 μl of the supernatant was used as template for the PCR, which was performed using the DreamTaq Green DNA Polymerase (Thermo Scientific) according to the manufacturer’s instructions.

### Spot dilution and liquid survival/growth inhibition assays

Spot dilution assays were performed as described previously [10]. For determination of H_2_O_2_ survival logarithmically growing cells in YPD were treated with the indicated concentrations of hydrogen peroxide for 2 hours at 30°C. Before and after treatment cells were diluted and plated on YPD plates. For the H_2_O_2_ survival shown in Fig 7D cells were grown overnight in minimal medium, diluted to OD_600_ of 0.2 and further incubated at 30°C for 5 hours. Cells were harvested by centrifugation at 1500 g for 3 minutes, resuspended at an OD_600_ of 0.5 and treated with 0.5 mM H_2_O_2_ for 2 hours. Before and after treatment cells were diluted and plated on YPD plates containing 1 mM glutathione. Colonies were counted after 3 days incubation at 30°C and viability was determined relative to the samples plated before H_2_O_2_ addition. To quantify growth inhibition by azole treatment cells were grown to logarithmic phase in SC medium at 30°C. Cultures were diluted to an OD_600_ of 0.01 in SC medium with or without voriconazole at the indicated concentrations. OD_600_ was determined after growth at 30°C for 18–24 hours. For *C*. *parapsilosis* cultures were incubated for 41 hours due to the slow growth rate of the *hat1*Δ/Δ mutant. Growth inhibition was calculated relative to untreated controls.

### RNA isolation, RT-qPCR analysis and RNA-seq

Cells were grown in YPD overnight to an OD_600_ of 1 at 30°C. For hydrogen peroxide treatment 1.6 mM H_2_O_2_ was added to the culture for the indicated period of time. RNA isolation and qPCR analysis was done as described previously [10]. For RT-qPCRs shown in Fig 8A, *RIP1* was used as reference gene [99]. All other RT-qPCRs were normalized to *PAT1* [110].

After RNA isolation, 10 μg total RNA were treated with DNase I (Thermo Scientific) and purified using the RNeasy MinElute Cleanup Kit (Qiagen). 5 μg DNase treated RNA were used for rRNA depletion with the RiboMinus Eukaryote System v2 (Life Technologies, Carlsbad, CA). rRNA depleted samples were fragmented using the NEBNext Magnesium RNA Fragmentation Module (New England Biolabs) and purified with the RNeasy MinElute Cleanup Kit (Qiagen). SuperScript III reverse transcriptase (Life Technologies, Carlsbad, CA) was used for first strand synthesis. Priming was done with 3 μg random hexamers (Life Technologies). Samples were purified using Mini Quick Spin Columns (Roche) and second strand synthesis was done with the NEBNext mRNA Second Strand Synthesis Module (New England Biolabs). Final purification of double stranded cDNA was done with the MinElute PCR Purification Kit (Qiagen). Samples were further processed and sequenced on a HiSeq 2000 instrument (Illumina) at the Next Generation Sequencing Facility (CSF NGS unit, http://www.csf.ac.at) of the Campus Vienna Biocenter. For both conditions five biological replicates for the wild-type as well as the *hat1*Δ/Δ strain and three biological replicates for the *cac2*Δ/Δ as well as the *rtt109*Δ/Δ mutants were sequenced.

Reads were mapped onto the Assembly 21 of the *C*. *albicans* genome (http://www.candidagenome.org) using NextGenMap [111]. Read counts were determined with HTSeq using the union mode [112] and a reference annotation (C_albicans_SC5314_version_A21-s02-m07-r10; http://www.candidagenome.org). The annotation of the coding sequence assembly was used as transcript coordinates. For short non-coding RNAs (tRNAs, snRNAs, snoRNAs and ncRNAs) 20bp up- and downstream of their chromosomal coordinates were added before mapping [113]. Differential expression analysis was done with edgeR [114]. Benjamini-Hochberg adjusted p-values were used to determine differentially regulated genes [115]. Venn diagrams were created using Venny 1.0 [116]. Gene ontology (GO) term enrichment was determined using the GO Term finder (http://www.candidagenome.org). Overlapping GO terms were merged manually.

### Chromatin immunoprecipitation

Chromatin immunoprecipitation was performed as described previously [113]. One mg whole cell extract was used per ChIP. For determination of histone density an antibody against the C-terminus of histone H3 was used (ab1791, Abcam). Detection of RNAPII was done with an antibody against the C-terminal domain (05–592, clone 8WG16, Millipore). To analyze the *CAT1* promoter region primers amplifying a fragment ranging from -315 to -163 with respect to the start codon were used. To determine enrichment within the *CAT1*, *GPX1* and *GST1* genes primers within these coding regions were used. Signals were normalized to an intergenic region on chromosome R.

### Enzymatic assays, immunoblotting and mass spectrometric analysis

To quantify catalase activity, cells were grown overnight to an OD_600_ of 1 at 30°C. For hydrogen peroxide treatment 1.6 mM H_2_O_2_ was added to the culture for one hour. Before and after treatment 20 ml culture were harvested at 1500 g for 3 min at 4°C and washed once with 20 ml cold H_2_O. Pellets were resuspended in 250 μl lysis buffer [50 mM Tris-HCl pH 7.5; 10% glycerol, complete protease inhibitor cocktail (Roche)] and an equal volume of glass beads (425–600mm, Sigma Aldrich) was added. Cells were lysed by shaking 5 times at 6 m s^-1^ for 30 s on a FastPrep instrument (MP Biomedicals). Extracts were cleared by centrifugation at 14000 g for 5 min at 4°C. Protein concentration in the extracts was determined by measuring absorption at 280 nm. For catalase activity measurement 5–40 μl whole cell extract were added to 3 ml of catalase assay buffer [384 mM Na_3_PO_4_; 0.015 mM Triton X-100 (Sigma Aldrich); 11.4 mM H_2_O_2_] and degradation of H_2_O_2_ was determined by measuring absorbance at 240 nm for up to 2 min. Catalase activity was calculated in μM H_2_O_2_ per minute per mg of whole cell extract as described previously [117] (ε = 43.6).

Glutathione peroxidase assays were performed as previously described [118] using some modifications. The lysis buffer used contained 50 mM Tris-HCl, pH 7.5, 150 mM NaCl, 0.5 g/100 ml Nonidet P-40 and complete protease inhibitor cocktail (Roche, Basel, Switzerland). Cell lysis was performed as described for the catalase assay. 10–50 μl cleared whole cell extract was used for the assay exactly as described in [118].

Sample preparation and western blot analysis were essentially carried out as described previously [119]. A MOI of 5:1 (fungi to macrophages) was used and samples were harvested after 30 min of interaction. Activated NADPH oxidase was detected using an antibody against the phosphorylated p40phox subunit (Cell Signaling 4311). A panERK antibody (BD 610123) was used as loading control.

Whole cell extracts for mass spectrometric analysis were prepared by lysing logarithmically growing cells in MS lysis buffer [10 mM Tris-HCl pH 7.5; complete protease inhibitor cocktail (Roche)] with a French press. Extracts were lyophilized, resuspended in 2 M urea and used for trypsin digestion followed by liquid chromatography—tandem mass spectrometry analysis on a LTQ Orbitrap Velos system (Thermo Scientific).

### Mouse strains and immune cells (bone marrow-derived macrophages and neutrophils)

For all experiments, 7–10 week old C57BL/6 wild-type mice were used. Isolation and cultivation of primary bone marrow-derived macrophages was done as described previously [26]. Isolation of bone marrow neutrophils and subsequent *C*. *albicans* survival assays were performed as described earlier [65]. A MOI of 1:10 (fungi to neutrophils) was used, and cells were harvested after a 1-hour interaction.

Mouse infections were carried out through lateral tail vein injections as described previously with some minor modifications [65]. Briefly, *C*. *albicans* strains were grown overnight to an OD_600_ of around 1, washed twice and finally resuspended in PBS. For infection, 1 x 10^5^ *Candida* cells per 21 g mouse body weight were injected via the lateral tail vein. For survival experiments, mice were monitored for 32 days. Analysis of fungal burdens in the kidneys at day 1, 3 and 7 post infection, as well as determination of serum urea levels was done exactly as described previously [65]. Statistical analysis was carried out using the Prism software (Graphpad Software Inc.).

### ROS and phagocytosis assays

ROS assays were done exactly as described previously [26]. The multiplicity of infection (MOI) for all ROS assays was 5:1 (fungi to immune cells). Phagocytosis assays were performed essentially as described with some modifications [119]. *C*. *albicans* cells were grown overnight to an OD_600_ of around 1, washed twice in PBS and stained with 10 mg ml^-1^ Alexa Fluor 488 (Life Technologies) in 100 mM HEPES buffer (pH 7.5) for 60 min at 30°C shaking in the dark. After staining cells were washed 3 times, resuspended in HEPES buffer and used for interaction with BMDMs. Stained *Candida* cells were added to macrophages and incubated for 45 min at 37°C and 5% CO_2_. Control reactions were kept on ice during the whole procedure. A MOI of 2:1 (fungi to macrophages) was used. Phagocytosis was terminated by chilling on ice. Plates remained on ice during subsequent detaching and fixation in 1% formaldehyde. Extracellular fluorescent *C*. *albicans* cells were quenched by addition of 0.4% trypan blue. Samples were subject to flow cytometry analysis with FL1-H on a FACSCalibur instrument (BD Biosciences).

## Supporting Information

S1 Fig Loss of Hat1 causes oxidative stress resistance. (A) Resistance to other stress conditions is unchanged in *hat1*Δ/Δ cells. CFW: Calcofluor White; CR: Congo Red; AmB: Amphotericin B; (B) Loss of Hat1 increases resistance to diamide. (C) Lack of proteins involved in DNA damage repair does not increase resistance to H_2_O_2_. (D) Deletion of *CAC2* causes increased tBOOH resistance. Loss of Rtt109 does not affect tBOOH resistance. (A-D) Fivefold serial dilutions of the indicated strains were spotted on agar plates containing the indicated substances and pictures were taken after incubation at 30°C for 3 days.
(TIF)

S2 Fig Deletion of *HAT1* causes upregulation of tRNAs and *CAT1*. (A) Treatment with 1.6 mM H_2_O_2_ does not kill *C*. *albicans*. Cells of the indicated strains were treated for 1 hour, plated and colonies counted after 3 days of incubation on YPD plates at 30°C to determine viability. Data are shown as mean + SD from two independent experiments. (B) H_2_O_2_ repressed ncRNAs are upregulated in the *hat1*Δ/Δ mutant upon peroxide treatment. Each dot corresponds to one ncRNA. The fold change in RNA expression between H_2_O_2_ treated wild-type and *hat1*Δ/Δ strains (y-axis) is plotted against the fold change between the wild-type without and with treatment (x-axis). Differentially expressed ncRNAs (fold change > = 2 and p-value <0.05) in the *hat1*Δ/Δ mutant are depicted in red. logFC: log2 fold change; (C) GO terms enriched among 1.5-fold upregulated proteins in logarithmically growing *hat1*Δ/Δ cells are shown. Expression levels were determined by mass spectrometric analysis as described in the Materials and Methods section. Fold changes relative to the wild-type were calculated using the spectra counts. The corresponding p-values for the enrichment (empty bars) and the percentage of proteins changed within the GO group (filled bars) are presented. The absolute number of regulated proteins within a GO group is presented in brackets. (D) Derepression of *CAT1* in logarithmically growing cells was detected by RT-qPCR. Transcript levels were normalized to the expression level of the reference gene (RG) *PAT1*. Data are shown as mean + SD from 3 independent experiments. (E) Increased *CAT1* induction levels were only observed for *hat1*Δ/Δ and *cac2*Δ/Δ cells, but not for cells lacking Rtt109. Cells were treated with 1.6 mM H_2_O_2_ for 30 min. Transcript levels were normalized to the expression level of *PAT1*. Data are shown as mean + SD from 3 independent experiments. (C-D) *P<0.05, **P<0.01, ***P<0.001 relative to the corresponding control (Student's t-test).
(TIF)

S3 Fig Loss of Hat1 causes reduced ROS accumulation upon interaction with neutrophils. (A) ROS levels were determined by measuring luminol-dependent chemiluminescence [relative luciferase units (RLU) min^-1^ per 1000 immune cells] in 2.5 min intervals during interaction of the indicated *C*. *albicans* strains with murine bone marrow neutrophils. One representative experiment is shown. Data were reproduced in two independent experiments. (B) Cells lacking Hat1 are phagocytosed at the same rate as the wild-type. Phagocytosis was quantified by measuring the fraction of BMDMs containing labelled *C*. *albicans* cells upon 45 min interaction at 37°C (5% CO_2_) by FACS. Control reactions were kept at 4°C. BMDMs without *C*. *albicans* were also included (Mphs only). One representative experiment is shown. Data were reproduced in two independent experiments. FL1-H: FITC intensity (C) NADPH oxidase is activated to the same extent upon infection with wild-type or *hat1*Δ/Δ cells. Activation of the NADPH oxidase in BMDMs upon 30 min interaction was determined by detection of the phosphorylated p40phox subunit. ERK levels served as loading control. An uninfected control was included (wo Ca). The asterisk marks a cross reaction. One representative experiment is shown. Data were reproduced in two independent experiments.
(TIF)

S4 Fig Reduced body weight loss upon infection with *hat1*Δ/Δ or *cac2*Δ/Δ cells. (A and B) Body weight of mice infected with the indicated *Candida* strains was determined daily until day 6 post infection (p.i.). Significance was determined relative to wild-type infected mice using Two-way ANOVA. ***P<0.001.
(TIF)

S5 Fig Deletion of *RTT109* in *S*. *cerevisiae* increases H_2_O_2_ resistance. Fivefold serial dilutions of the indicated strains were spotted on agar plates containing the indicated substances and pictures were taken after incubation at 30°C for 3 days.
(TIF)

S1 Table RNA-Seq results. (XLSX)

S1 Text Fungal strains, plasmids and oligonucleotides used in this study. Table A. Fungal strains used in this study. Table B. Plasmids used in this study. Table C. Oligonucleotides used in this study.
(DOCX)
