# Supplementary figures and images for: The Candida albicans Histone Acetyltransferase Hat1 Regulates Stress Resistance and Virulence via Distinct Chromatin Assembly Pathways
Source: PLoS Pathog. 2015 Oct 16;11(10):e1005218. doi: 10.1371/journal.ppat.1005218 (PMC4608838; doi:10.1371/journal.ppat.1005218)

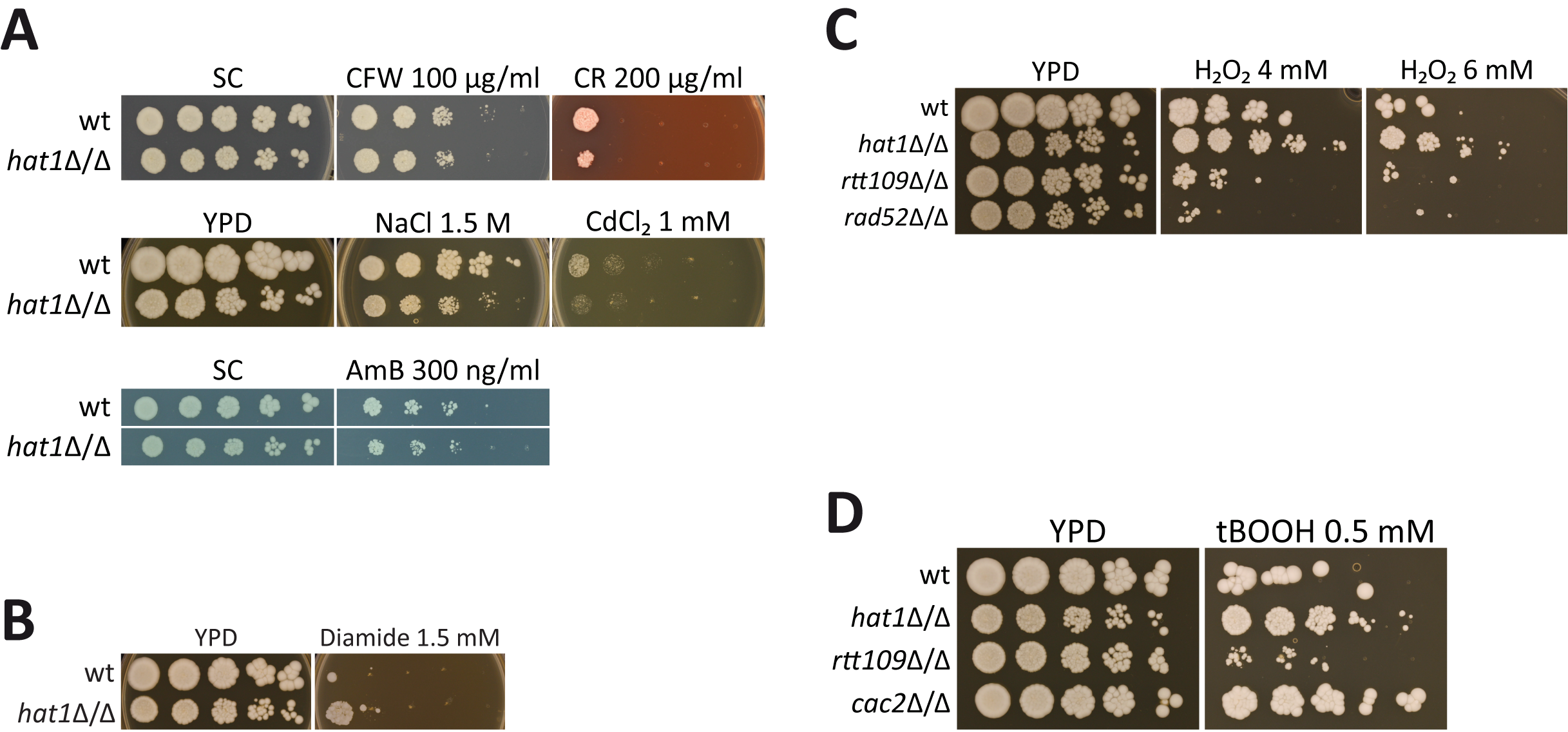

Supplement: S1 Fig — (A) Resistance to other stress conditions is unchanged in hat1Δ/Δ cells. CFW: Calcofluor White; CR: Congo Red; AmB: Amphotericin B; (B) Loss of Hat1 increases resistance to diamide. (C) Lack of proteins involved in DNA damage repair does not increase resistance to H2O2. (D) Deletion of CAC2 causes increased tBOOH resistance. Loss of Rtt109 does not affect tBOOH resistance. (A-D) Fivefold serial dilutions of the indicated strains were spotted on agar plates containing the indicated substances and pictures were taken after incubation at 30°C for 3 days. (TIF) [file ppat.1005218.s001.tif]

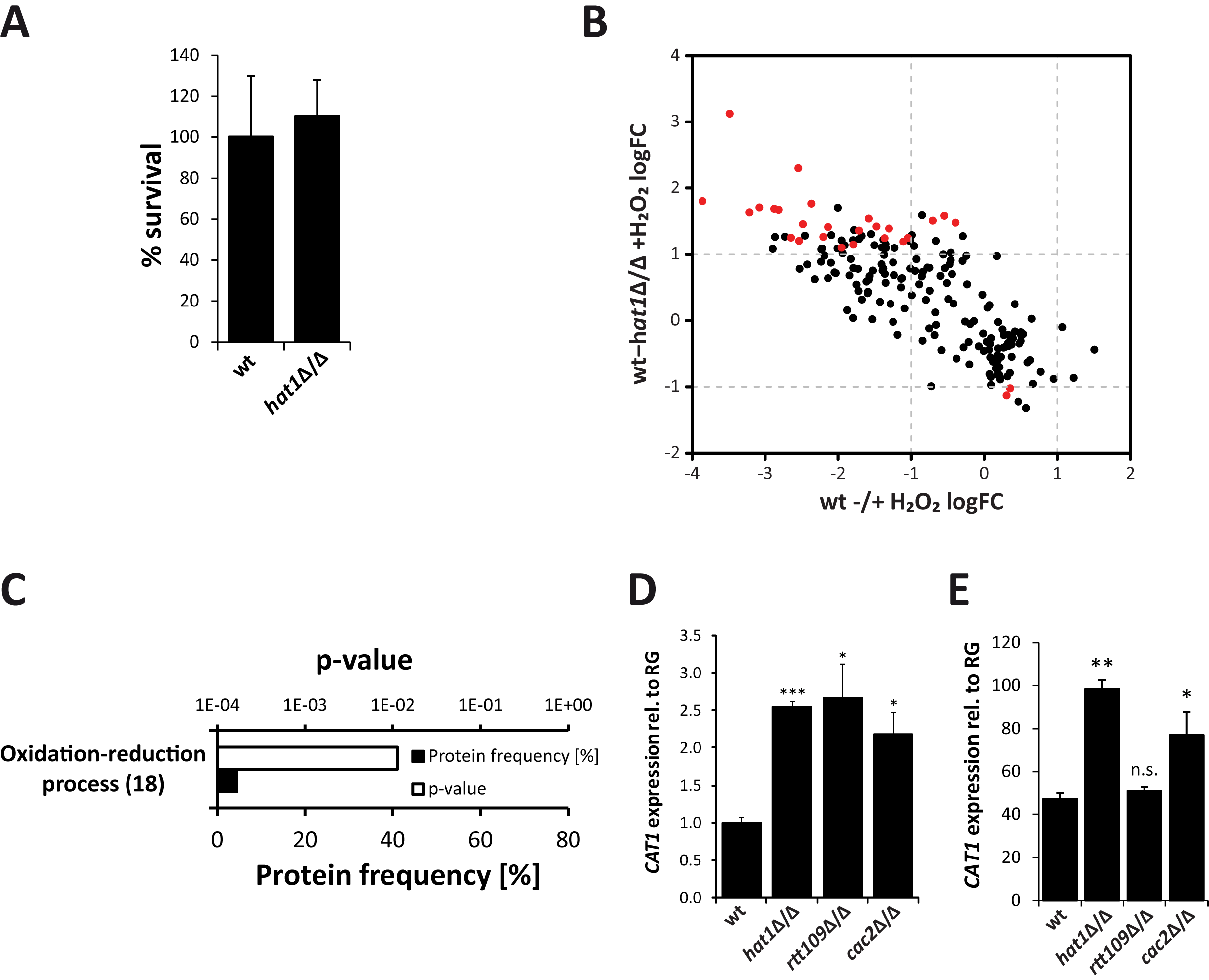

Supplement: S2 Fig — (A) Treatment with 1.6 mM H2O2 does not kill C. albicans. Cells of the indicated strains were treated for 1 hour, plated and colonies counted after 3 days of incubation on YPD plates at 30°C to determine viability. Data are shown as mean + SD from two independent experiments. (B) H2O2 repressed ncRNAs are upregulated in the hat1Δ/Δ mutant upon peroxide treatment. Each dot corresponds to one ncRNA. The fold change in RNA expression between H2O2 treated wild-type and hat1Δ/Δ strains (y-axis) is plotted against the fold change between the wild-type without and with treatment (x-axis). Differentially expressed ncRNAs (fold change > = 2 and p-value <0.05) in the hat1Δ/Δ mutant are depicted in red. logFC: log2 fold change; (C) GO terms enriched among 1.5-fold upregulated proteins in logarithmically growing hat1Δ/Δ cells are shown. Expression levels were determined by mass spectrometric analysis as described in the Materials and Methods section. Fold changes relative to the wild-type were calculated using the spectra counts. The corresponding p-values for the enrichment (empty bars) and the percentage of proteins changed within the GO group (filled bars) are presented. The absolute number of regulated proteins within a GO group is presented in brackets. (D) Derepression of CAT1 in logarithmically growing cells was detected by RT-qPCR. Transcript levels were normalized to the expression level of the reference gene (RG) PAT1. Data are shown as mean + SD from 3 independent experiments. (E) Increased CAT1 induction levels were only observed for hat1Δ/Δ and cac2Δ/Δ cells, but not for cells lacking Rtt109. Cells were treated with 1.6 mM H2O2 for 30 min. Transcript levels were normalized to the expression level of PAT1. Data are shown as mean + SD from 3 independent experiments. (C-D) *P<0.05, **P<0.01, ***P<0.001 relative to the corresponding control (Student's t-test). (TIF) [file ppat.1005218.s002.tif]

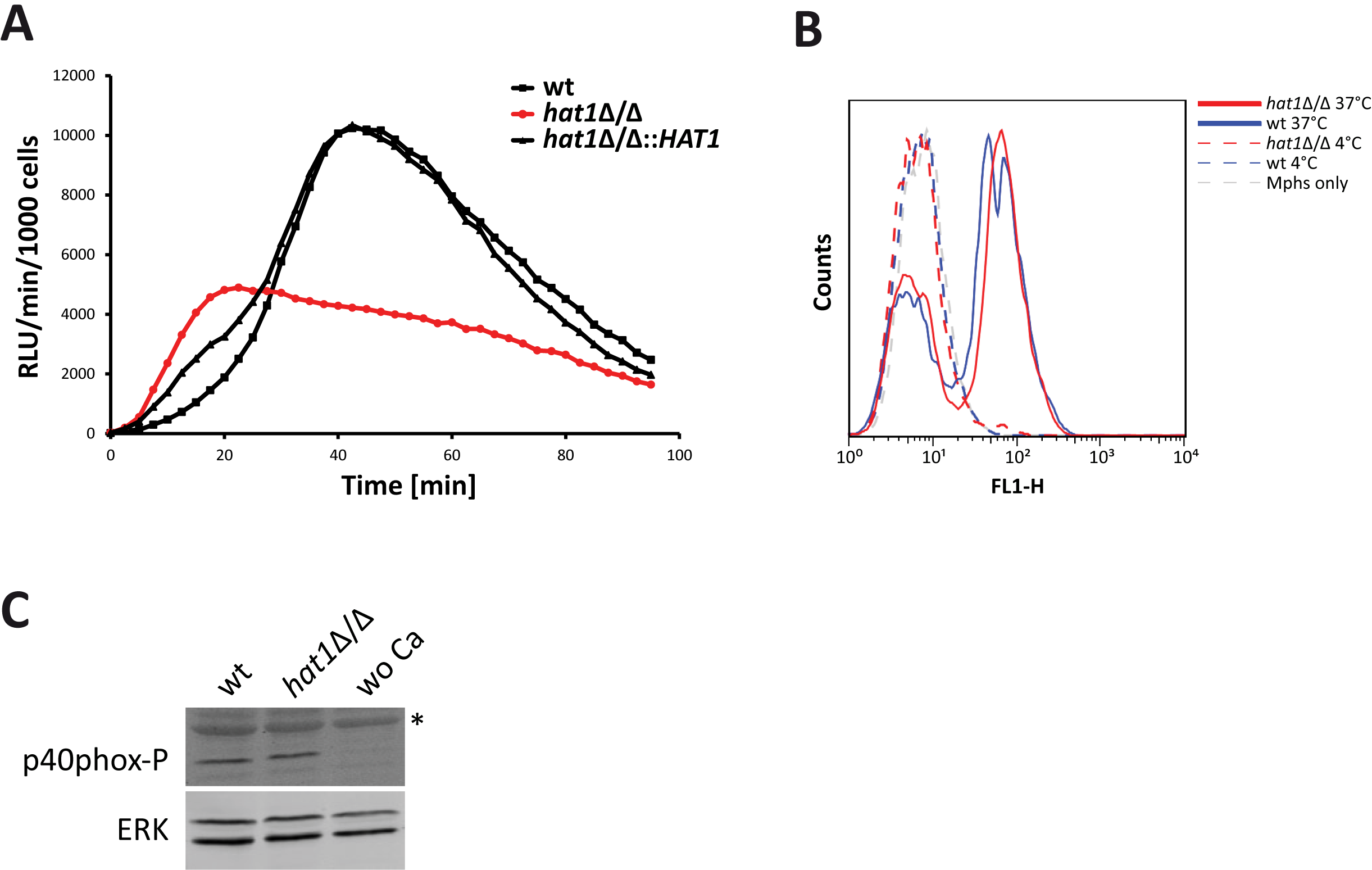

Supplement: S3 Fig — (A) ROS levels were determined by measuring luminol-dependent chemiluminescence [relative luciferase units (RLU) min-1 per 1000 immune cells] in 2.5 min intervals during interaction of the indicated C. albicans strains with murine bone marrow neutrophils. One representative experiment is shown. Data were reproduced in two independent experiments. (B) Cells lacking Hat1 are phagocytosed at the same rate as the wild-type. Phagocytosis was quantified by measuring the fraction of BMDMs containing labelled C. albicans cells upon 45 min interaction at 37°C (5% CO2) by FACS. Control reactions were kept at 4°C. BMDMs without C. albicans were also included (Mphs only). One representative experiment is shown. Data were reproduced in two independent experiments. FL1-H: FITC intensity (C) NADPH oxidase is activated to the same extent upon infection with wild-type or hat1Δ/Δ cells. Activation of the NADPH oxidase in BMDMs upon 30 min interaction was determined by detection of the phosphorylated p40phox subunit. ERK levels served as loading control. An uninfected control was included (wo Ca). The asterisk marks a cross reaction. One representative experiment is shown. Data were reproduced in two independent experiments. (TIF) [file ppat.1005218.s003.tif]

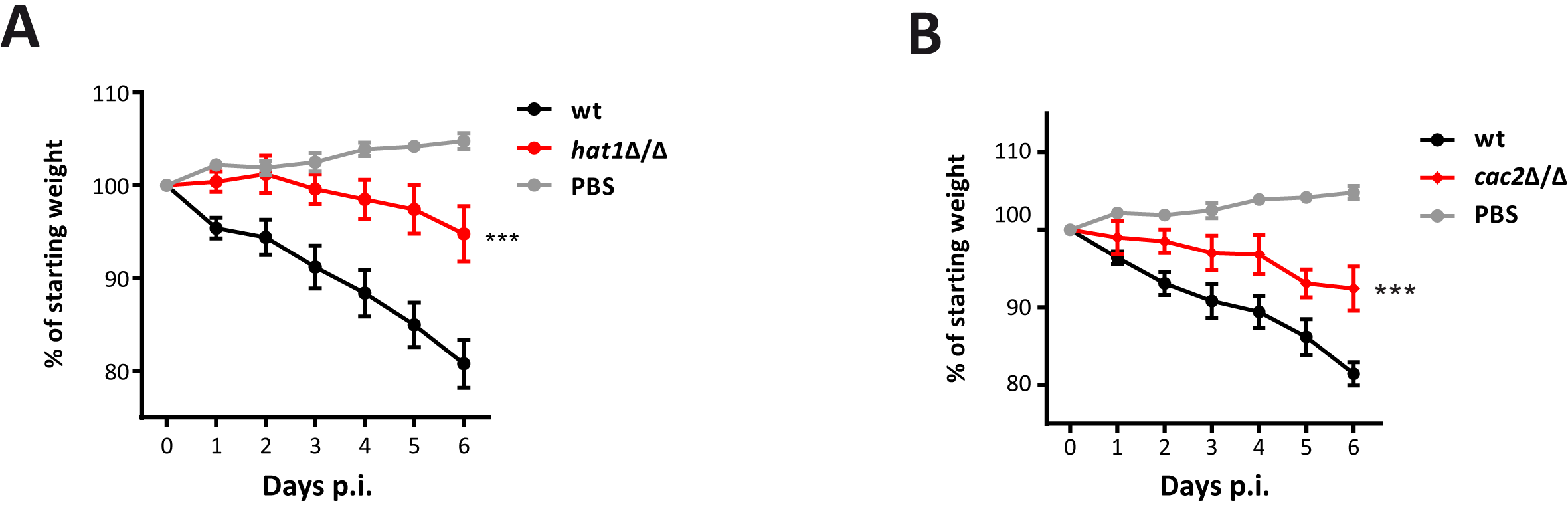

Supplement: S4 Fig — (A and B) Body weight of mice infected with the indicated Candida strains was determined daily until day 6 post infection (p.i.). Significance was determined relative to wild-type infected mice using Two-way ANOVA. ***P<0.001. (TIF) [file ppat.1005218.s004.tif]

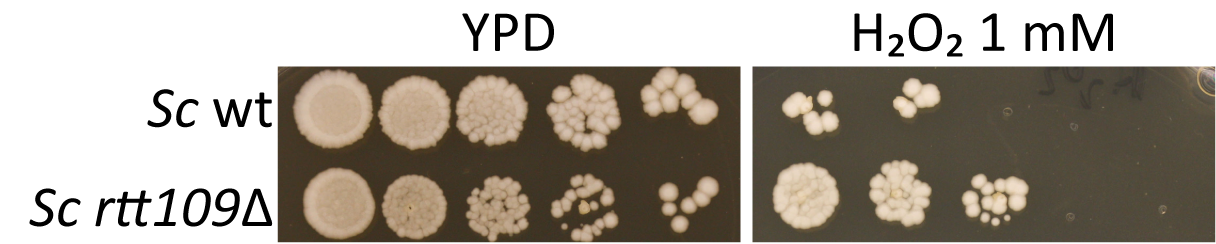

Supplement: S5 Fig — Fivefold serial dilutions of the indicated strains were spotted on agar plates containing the indicated substances and pictures were taken after incubation at 30°C for 3 days. (TIF) [file ppat.1005218.s005.tif]
