# Supplementary material for: The Candida albicans Histone Acetyltransferase Hat1 Regulates Stress Resistance and Virulence via Distinct Chromatin Assembly Pathways
Source: PLoS Pathog. 2015 Oct 16;11(10):e1005218. doi: 10.1371/journal.ppat.1005218 (PMC4608838; doi:10.1371/journal.ppat.1005218)
Supplement: S1 Text — Table A. Fungal strains used in this study. Table B. Plasmids used in this study. Table C. Oligonucleotides used in this study. (DOCX) [file ppat.1005218.s007.docx]

**S1 Text. Fungal strains, plasmids and oligonucleotides used in this study.**

**Table A. Fungal strains used in this study.**

**Table B. Plasmids used in this study.**

**Table C. Oligonucleotides used in this study.**

[[1-13](#_ENREF_1)]

**Supplemental References**

1. Gillum AM, Tsay EY, Kirsch DR. Isolation of the *Candida albicans* gene for orotidine-5'-phosphate decarboxylase by complementation of *S. cerevisiae* ura3 and *E. coli* pyrF mutations. Mol Gen Genet. 1984 198: 179-182.

2. Tscherner M, Stappler E, Hnisz D, Kuchler K. The histone acetyltransferase Hat1 facilitates DNA damage repair and morphogenesis in *Candida albicans*. Mol Microbiol. 2012 Dec; 86: 1197-1214.

3. Noble SM, Johnson AD. Strains and strategies for large-scale gene deletion studies of the diploid human fungal pathogen *Candida albicans*. Eukaryot Cell. 2005 Feb; 4: 298-309.

4. Al-Rawi N, Laforce-Nesbitt SS, Bliss JM. Deletion of *Candida albicans SPT6* is not lethal but results in defective hyphal growth. Fungal Genet Biol. 2010 Apr; 47: 288-296.

5. Brachmann CB, Davies A, Cost GJ, Caputo E, Li J, et al. Designer deletion strains derived from *Saccharomyces cerevisiae* S288C: a useful set of strains and plasmids for PCR-mediated gene disruption and other applications. Yeast. 1998 Jan 30; 14: 115-132.

6. Kitada K, Yamaguchi E, Arisawa M. Cloning of the *Candida glabrata TRP1* and *HIS3* genes, and construction of their disruptant strains by sequential integrative transformation. Gene. 1995 Nov 20; 165: 203-206.

7. Schwarzmüller T, Ma B, Hiller E, Istel F, Tscherner M, et al. Systematic Phenotyping of a Large-Scale *Candida glabrata* Deletion Collection Reveals Novel Antifungal Tolerance Genes. PLoS Pathog. 2014 Jun; 10: e1004211.

8. Leupold U. The inheritance of homothally and heterothally in *Schizosaccharomyces pombe*. Compt Rend Trav Lab Carlsberg Ser Physiol. 1950 24: 381–480.

9. Benson LJ, Phillips JA, Gu Y, Parthun MR, Hoffman CS, et al. Properties of the type B histone acetyltransferase Hat1: H4 tail interaction, site preference, and involvement in DNA repair. J Biol Chem. 2007 Jan 12; 282: 836-842.

10. Gacser A, Salomon S, Schafer W. Direct transformation of a clinical isolate of *Candida parapsilosis* using a dominant selection marker. FEMS Microbiol Lett. 2005 Apr 1; 245: 117-121.

11. Krauke Y, Sychrova H. Cnh1 Na(+) /H(+) antiporter and Ena1 Na(+) -ATPase play different roles in cation homeostasis and cell physiology of *Candida glabrata*. FEMS Yeast Res. 2011 Feb; 11: 29-41.

12. Reuss O, Vik A, Kolter R, Morschhäuser J. The SAT1 flipper, an optimized tool for gene disruption in *Candida albicans*. Gene. 2004 Oct 27; 341: 119-127.

13. Hnisz D, Majer O, Frohner IE, Komnenovic V, Kuchler K. The Set3/Hos2 histone deacetylase complex attenuates cAMP/PKA signaling to regulate morphogenesis and virulence of *Candida albicans*. PLoS Pathog. 2010 May; 6: e1000889.
